# Functionally dominant hotspot mutations of mitochondrial ribosomal RNA genes in cancer

**DOI:** 10.1038/s41588-025-02374-0

**Published:** 2025-11-03

**Authors:** Sonia Boscenco, Jacqueline Tait-Mulder, Minsoo Kim, Cerise Tang, Tricia Park, Flora McNulty, Sergio Lilla, Sara Zanivan, Alejandro Huerta-Uribe, Benan Nalbant, Mark Zucker, David Sumpton, Geoffray Monteuuis, Christopher B. Jackson, Wei Wei, Patrick F. Chinnery, Ronan Chaligne, Caleb A. Lareau, Ed Reznik, Payam A. Gammage

**Affiliations:** 1 Computational Oncology, Memorial Sloan Kettering Cancer Center, New York, NY USA; 2 Department of Physiology, Biophysics, and Systems Biology, Weill Cornell Medicine, New York, NY USA; 3 Cancer Research UK Scotland Institute, Glasgow, UK; 4 School of Cancer Sciences, University of Glasgow, Glasgow, UK; 5 Department of Experimental Therapeutics, University of Texas MD Anderson Cancer Center, Houston, TX USA; 6 Department of Computational and Systems Biology, Memorial Sloan Kettering Cancer Center, New York, NY USA; 7 Department of Pathology, New York University, New York, NY USA; 8 Department of Biochemistry and Developmental Biology, University of Helsinki, Helsinki, Finland; 9 Department of Clinical Neuroscience, School of Clinical Medicine, University of Cambridge, Cambridge, UK; 10 Medical Research Council Mitochondrial Biology Unit, University of Cambridge, Cambridge, UK

**Keywords:** Cancer, Genetics

## Abstract

The vast majority of recurrent somatic mutations arising in tumors affect protein-coding genes in the nuclear genome. Here, through population-scale analysis of 14,106 whole tumor genomes, we report the discovery of highly recurrent mutations affecting both the small (12S, *MT-RNR1*) and large (16S, *MT-RNR2*) mitochondrial RNA subunits of the mitochondrial ribosome encoded within mitochondrial DNA (mtDNA). Compared to non-hotspot positions, mitochondrial rRNA hotspots preferentially affected positions under purifying selection in the germline and demonstrated structural clustering within the mitoribosome at mRNA and tRNA interacting positions. Using precision mtDNA base editing, we engineered models of an exemplar *MT-RNR1* hotspot mutation, m.1227G>A. Multimodal profiling revealed a heteroplasmy-dependent decrease in mitochondrial function and loss of respiratory chain subunits from a heteroplasmic dosage of ~10%. Mutation of conserved positions in ribosomal RNA that disrupt mitochondrial translation therefore represent a class of functionally dominant, pathogenic mtDNA mutations that are under positive selection in cancer genomes.

## Abstract (web summary)

Analysis of 14,106 tumor genomes highlights recurrent mutations in mitochondrial ribosomal RNA encoded within the mitochondrial genome. Mutations occur at hotspot positions and are under strong purifying selection in the germline.

## Main

Both the nuclear and mitochondrial genomes are targets of recurrent somatic mutations (‘hotspots’) to protein-coding genes and non-protein-coding genetic elements in cancer^1–3^. mtDNA encodes its own translational machinery, including two mitochondrial ribosomal RNAs (mt-rRNAs) and 22 mitochondrial transfer RNAs (mt-tRNAs). Mutations to mt-tRNA and mt-rRNA can have profound adverse effects, ultimately disrupting the translation of the 13 protein-coding genes within mtDNA essential for carrying out oxidative phosphorylation, without directly compromising translation of nuclear-DNA-encoded genes. As a result, although mutations to nuclear-encoded tRNAs are not observed in cancers, recurrent and highly pathogenic mutations to certain mt-tRNAs have been observed (for example, m.3244G>A in Hurthle cell carcinoma^4,5^) and evidence suggests that specific secondary structural elements of mitochondrial tRNAs are recurrently disrupted across cancers^1^. In contrast, there is no existing evidence that the highly conserved mt-rRNAs are under positive selection for mutations in cancer. Pathogenic germline mutations of mt-rRNA have been observed anecdotally, and at low frequency in heterogeneous clinical sequencing data^6,7^, but with the exception of two rare germline polymorphisms associated with antibiotic-induced deafness^8,9^, mt-rRNA variants are not an established cause of pathology in humans.

As mtDNA is a multicopy genome, a key determinant of mtDNA mutation-associated phenotypes is allelic dosage of the mutation, known as heteroplasmy^10,11^. In hereditary mitochondrial disease, where mtDNA mutations have been studied most closely, penetrance of clinical phenotypes is complex, demonstrating mutant allele-level, tissue-level and heteroplasmy-level dependency. In general, heteroplasmic mutation burden and disease severity are correlated positively, with most heteroplasmic mtDNA disease manifesting clinically at heteroplasmies in excess of ~60% (ref. ^12^). Similarly, high heteroplasmy truncating mtDNA mutations observed commonly in cancer elicit a lineage-agnostic transcriptional phenotype, whereas low-heteroplasmy truncating variants affecting identical alleles elicit heterogeneous or no phenotypes, implying that pathogenic mtDNA mutations in cancer act in a functionally recessive manner^1^. With the exception of a single case report^13^ of a 25% heteroplasmy variant affecting the central base of an mt-tRNA anticodon, this functionally recessive classification is consistent across all classes of mitochondrial disease-associated mtDNA mutation to date.

The predominant focus of population-scale cancer genomics has been the nuclear genome; consequently, whereas hundreds of thousands of tumors have now been profiled by targeted sequencing focused on nuclear-DNA-encoded cancer genes, the largest existing studies of somatic alterations to mtDNA are around one to two orders of magnitude smaller. Despite this, previous efforts have identified highly recurrent somatic truncating mutations to mtDNA genes in cancer^1^ that have since been shown to drive divergent tumor biology and therapeutic sensitivity^14^. Here, we studied 14,106 tumors, profiled by whole-genome sequencing (WGS) as part of the 100,000 Genomes Project by Genomics England (GEL)^15^, with the goal of comprehensively delineating patterns of selection for mtDNA mutations in cancer.

## Results

### Landscape of hotspot mutations to mtDNA

**Extended Data Fig. 1:**
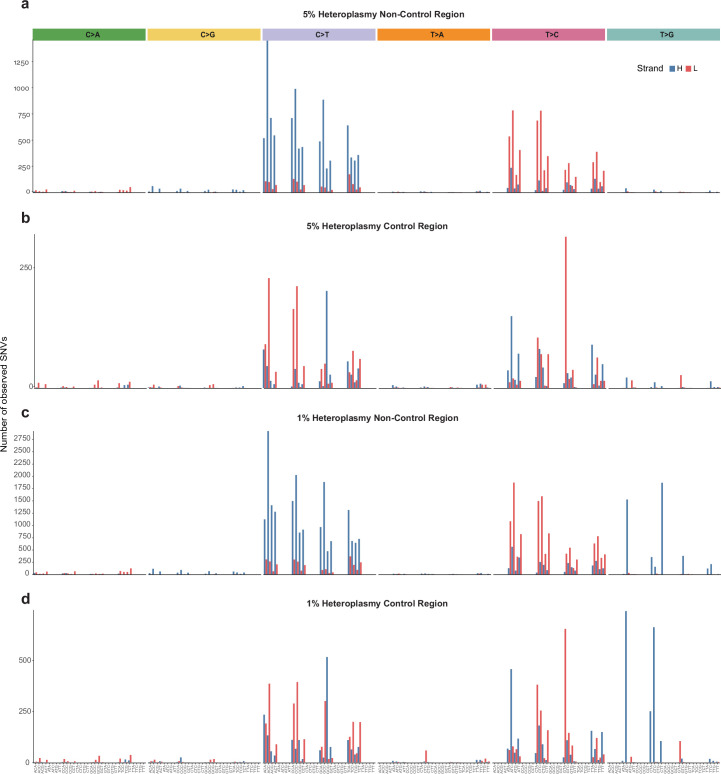
Mutational signatures of variants detected in tumor mtDNA. **a-b**. Mutation signature for all mtDNA SNV mutations using a 5% tumour heteroplasmy threshold for both control and non-control regions. **c-d**. Same as **a**-**b** but utilizing a 1% heteroplasmy threshold.

**Extended Data Fig. 2:**
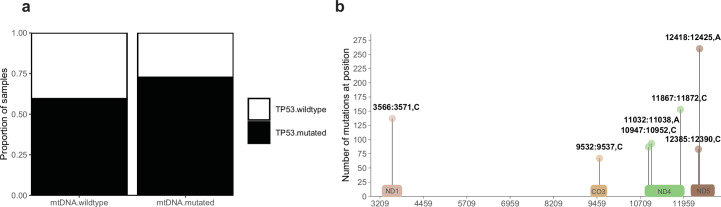
Co-mutational patterns and mtDNA indel hotspots. **a**. Co-mutation patterns between any type of mtDNA alteration, and TP53 mutations in breast cancer (q = 0.031, OR = 1.43, chi-squared test). **b**. Lollipop plot illustrating indel hotspot mutations at homopolymeric regions. X-axis represents mitochondrial genome genomic position.

To study selection for somatic single nucleotide variants (SNVs) in tumor mtDNA, we identified somatic mtDNA variants across primary tumors from the GEL cohort (*n* = 14,106). The sheer magnitude of the sample size in this dataset, in conjunction with the high coverage depth of mtDNA reads (mean = 15,919×), enabled high-confidence identification of mtDNA variants to tumor heteroplasmies of 5%. In total, we identified 18,104 SNVs and 2,222 indels (Supplementary Table 1), consistent with previously reported estimates of approximately one somatic mutation in every two tumors^1–3^. The identified mutations exhibited a strand-specific mutation signature, with a predominant occurrence of C>T mutations on the heavy strand and T>C on the light strand in the non-control region that was reversed in the control region^2^ (Extended Data Fig. 1a,b). These mutations occur largely independently of known nuclear driver mutations, with the exception of a co-occurrence of *TP53* mutation and mtDNA mutations in breast cancer (*Q* = 0.031, odds ratio (OR) = 1.43, chi-squared test) (Extended Data Fig. 2a and Supplementary Table 4).

**Fig. 1:**
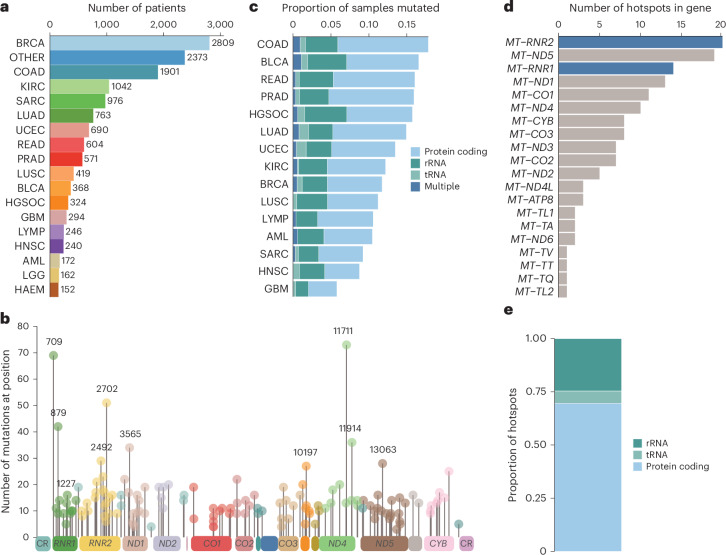
Landscape of mtDNA hotspot mutations across cancers. **a**, Summary of number of patients across tissue types. **b**, Frequency of hotspot mutations across positions of the entire mitochondrial genome. Mitochondrial genes are annotated at the bottom. **c**, Summary of the proportion of patients carrying a hotspot mutation across specific cancer types. Each sample is classified as either harboring a protein-coding, rRNA, tRNA or multiple hotspot mutation(s). **d**, Distribution of the frequency of hotspots across the mitochondrial genes. The rRNA genes are highlighted in blue. **e**, Classification of hotspot mutations as either occurring in a protein-coding region, tRNA or rRNA region.

Although the landscape of hotspot mutations in nuclear-DNA-encoded genes is relatively well described, a lack of statistical power has impeded an analogous, comprehensive analysis in mtDNA^16,17^. To do so, we applied a hotspot detection algorithm that identified mtDNA loci demonstrating a mutation burden in excess of the expected background mutational processes in mtDNA (Methods). In total, we recovered 138 unique statistically significant SNV hotspots (*Q* < 0.05) across 21 tumor lineages (Fig. 1a,b and Supplementary Table 2) and seven indel hotspots occurring at homopolymeric sites in complex I genes, as previously described by our group (Extended Data Fig. 2b and Supplementary Table 3). SNV hotspots affected diverse genetic elements, including protein-coding genes (*n* = 96 hotspots, 12 of 13 distinct genes), tRNA genes (*n* = 8 hotspots, 6 of 22 distinct genes) and rRNA genes (*n* = 34 hotspots, 2 of 2 genes) (Fig. 1b,c,e). We investigated the possibility that these hotspots reflected background somatic mutational processes recently reported in the mtDNA of non-neoplastic cells^18^. In total, 7 of 138 SNV hotspots, and four of seven indel hotspots, were identified as hotspots in non-neoplastic cells. Thus, whereas previously described indel hotspots are often observed as somatic mutations in nonmalignant cells, the vast majority of SNV hotspots are specific to cancer.

### mt-rRNA is the target of recurrent somatic mutations

**Extended Data Fig. 3:**
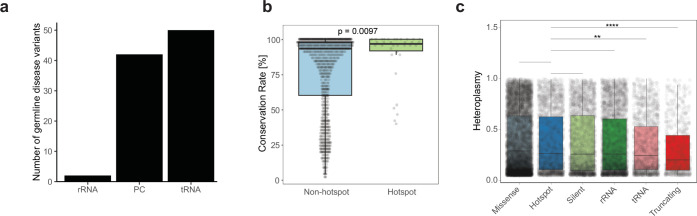
Differential features of germline and somatic mutations to mtDNA. **a**. Incidence of known germline-disease causing variants, as reported in the MITOMAP database. Of note, the two rRNA variants listed are associated with hearing loss upon treatment with aminoglycoside antibiotics, but are not otherwise associated with pathology. **b**. Comparison of evolutionary conservation rate, as estimated by the MITOMAP database utilizing 45 distinct species, between rRNA non-hotspot and hotspot mutation positions. P-value = 0.0097, two-sided Wilcoxon test. Boxplots indicate median and interquartile range; error bars, s.e.m. **c**. Comparison of heteroplasmy across mutation classes for all SNV mutations. P = 0.0081 and p < 4.3e-6 for **, and ****, respectively, two-sided Wilcoxon test. Boxplots indicate median and interquartile range; error bars, S.E.M. Significance assessed from Wilcoxon test.

**Extended Data Fig. 4:**
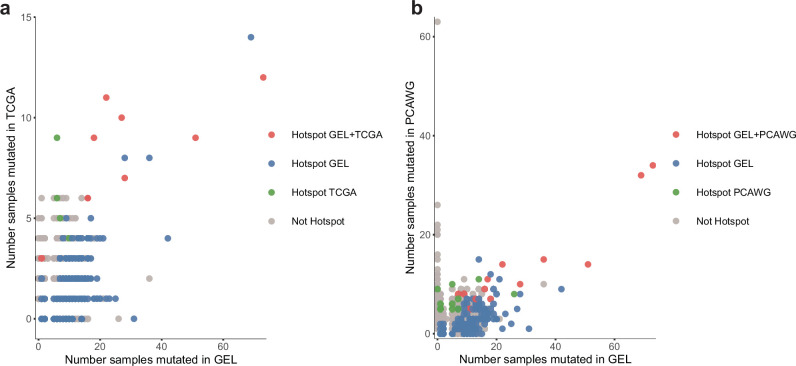
Recurrence of single nucleotide variants in tumor mtDNA is corroborated in independent cohorts. **a**. Incidence of mutations shared across the GEL and TCGA cohorts. Mutations that were not identified as hotspots are marked in grey, while hotspots are colored depending if the hotspot is in both cohorts or is unique. **b**. Same as in (**a**) but across the GEL and the PCAWG cohorts.

Unexpectedly, the rRNA-encoding genes *MT-RNR1* and *MT-RNR2* emerged as top ranked genes in terms of the number of somatic hotspot mutations, second only to *MT-ND5* (Fig. 1d). In total, ~4% of all tumors harbored at least one rRNA hotspot mutation. Somatic mutations to the ribosomal genes (as opposed to the other genetic elements in mtDNA) have been associated with cancer only anecdotally^19^. Functional mutations to *MT-RNR1* and *MT-RNR2* are similarly uncommon in the germline mitochondrial disease literature, where only 2 of 94 of known pathogenic germline variants identified in the MITOMAP database^19^ (m.1555A>G and m.1494C>T, associated with aminoglycoside-induced hearing loss, but not bona fide mitochondrial disease) arise in either rRNA gene, compared to 50 of 94 variants in the mitochondrial tRNAs (Extended Data Fig. 3a). To corroborate that the detection of rRNA hotspot variants was not an artifact of the GEL cohort or of our approach to variant calling, we examined the prevalence of hotspots in two other cohorts (The Cancer Genome Atlas Program (TCGA), a whole-exome sequencing cohort where we previously called somatic mtDNA mutations, and Pan-Cancer Analysis of Whole Genomes Cohort (PCAWG)—an WGS cohort where mtDNA mutations were called by the International Cancer Genome Consortium). Although comparatively smaller in sample size than GEL, the recurrence of mutant alleles in both TCGA and PCAWG was highly correlated with the analogous incidence in GEL (*P* = 0.027, *R* = 0.703, and *P* = 9 × 10^−7^, *R* = 0.94, respectively) (Extended Data Fig. 4a,b), confirming that rRNA mutations arising at specific loci are observed recurrently across clinically diverse sequencing cohorts, and suggesting that the failure to detect these alleles as recurrent in previous analyses arose from inadequate statistical power.

### mt-rRNA hotspots occur at loci under increased purifying selection

**Fig. 2:**
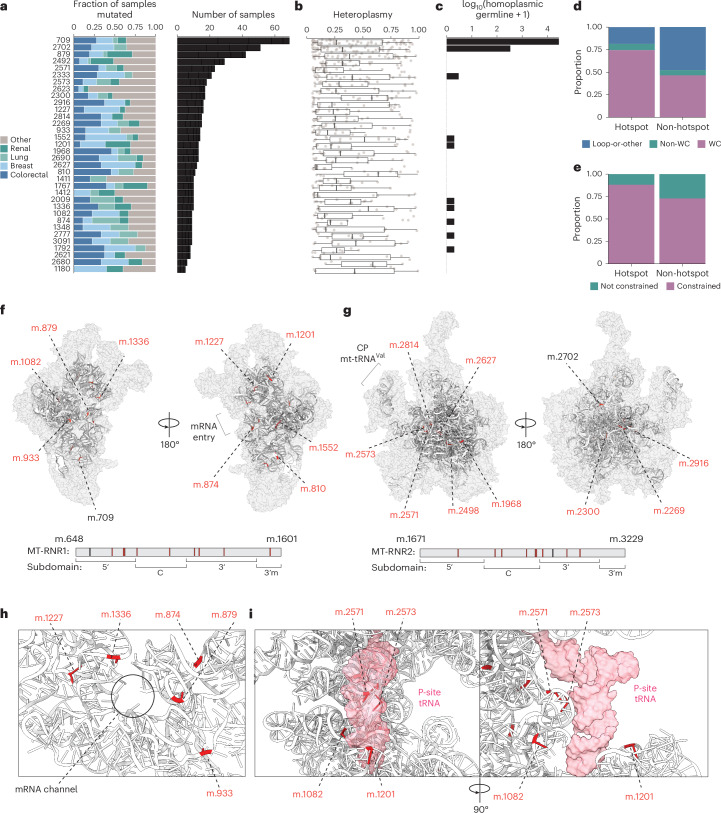
rRNA hotspot loci are under strong purifying selection and are clustered within the mitoribosome. **a**, Proportion of samples that harbor each rRNA hotspot across top four most common tissue lineages and the raw number of samples harboring each mutation. **b**, Heteroplasmy distributions across hotspot mutations. Boxplots indicate median and interquartile range; error bars, s.e.m. **c**, Log-scaled number of homoplasmic germline variants in the HelixMTDb. **d**, Proportion of positions occupying each possible secondary structure configuration at rRNA hotspot and non-hotspot mutation positions. Two-sided Fisher test *P* = 0.0040. WC, Watson–Crick. **e**, Proportion of rRNA hotspot and non-hotspot mutations that occupy positions homoplasmically mutated in the germline (as established by HelixMTDb). Two-sided Fisher test *P* = 0.0491. **f**,**g**, Location of the ten most recurrent hotspot mutations across mtSSU (**f**) and mtLSU (**g**). **h**, Cluster of hotspot mutations proximal to mtSSU mRNA channel. **i**, Cluster of hotspot mutations across *MT-RNR1* and *MT-RNR2* proximal to P-site tRNA. Structure adapted from ref. ^21^. PDB: 7qi5.

We investigated the broad tissue and genomic contexts under which rRNA hotspots arise. Unlike previously described protein-truncating hotspots^1^, rRNA hotspot mutations lacked tissue specificity (Fig. 2a; Methods) and did not exhibit characteristically elevated heteroplasmy mutations (relative to silent mutations) (Fig. 2b and Extended Data Fig. 3c). We investigated the extent of germline selection at rRNA hotspot loci. We observed that rRNA hotspots arose preferentially at germline-constrained positions (that is, positions with nearly no reported homoplasmic germline polymorphisms; Methods) as compared to non-hotspot rRNA alleles (*P* = 0.049, OR = 2.77, Fisher test) (Fig. 2e). Among the 34 rRNA hotspot alleles, only ten were at positions exhibiting any homoplasmic germline polymorphisms among the ~200,000 people profiled in HelixMTDb^20^, and only 3 of 34 exhibited more than one homoplasmic polymorphism (minor allele frequency greater than 1 × 10^−5^) (Fig. 2c; Methods). These data indicated that the genetic loci at which rRNA hotspots arose in cancer undergo strong purifying selection in the germline, and therefore indicated that rRNA hotspot alleles are functionally disruptive. To further assess the extent of purifying selection at hotspot positions, we used comparative genomics data from the MitoMAP database to quantify the evolutionary conservation rate of each position across 45 species. Hotspot rRNA alleles affected loci with elevated conservation rates as compared to non-hotspot rRNA mutations (*P* = 0.0097, Wilcoxon test; Extended Data Fig. 3b). Consistent with this, we observed that rRNA hotspots affected significantly more Watson–Crick pairing sites compared to non-hotspot rRNA mutations (*P* = 0.0040, Fisher test, OR = 1.47), implying that mutations at rRNA hotspots were at an increased likelihood to disrupt rRNA secondary structure (Fig. 2d).

Structural analysis of the 20 most recurrent rRNA hotspots across the small and large mitochondrial ribosomal subunits (mtSSU and mtLSU, respectively)^21^ (Fig. 2f,g) revealed clustering of mutated positions at specific structural features for 9 of 20 hotspots. A cluster of variants from both MT-RNR1 and MT-RNR2 was located in tRNA-contacting or proximal positions within the A-site (m.1227) and P-site (m.1082, m.1201, m.2571, m.2573), with a further cluster of variants (m.874, m.879, m.933, m.1336) at the entrance to the mRNA channel within mtSSU (Fig. 2h,i). Variants m.709 and m.2702 are located in peripheral regions not known to directly influence catalytic activity of the mitoribosome, consistent with the elevated rate of germline polymorphism at these loci and, therefore, probably more subtle phenotypic impacts. No hotspot variants were present in the decoding or peptidyl transferase centers of the mitoribosome. Taken together, these analyses indicate that somatic hotspots in *MT-RNR1* and *MT-RNR2* occur at loci under strong purifying selection in the germline, with structural features likely to result in loss-of-function.

### Functional impact of *MT-RNR1* mutation in human cancer cells

**Fig. 3:**
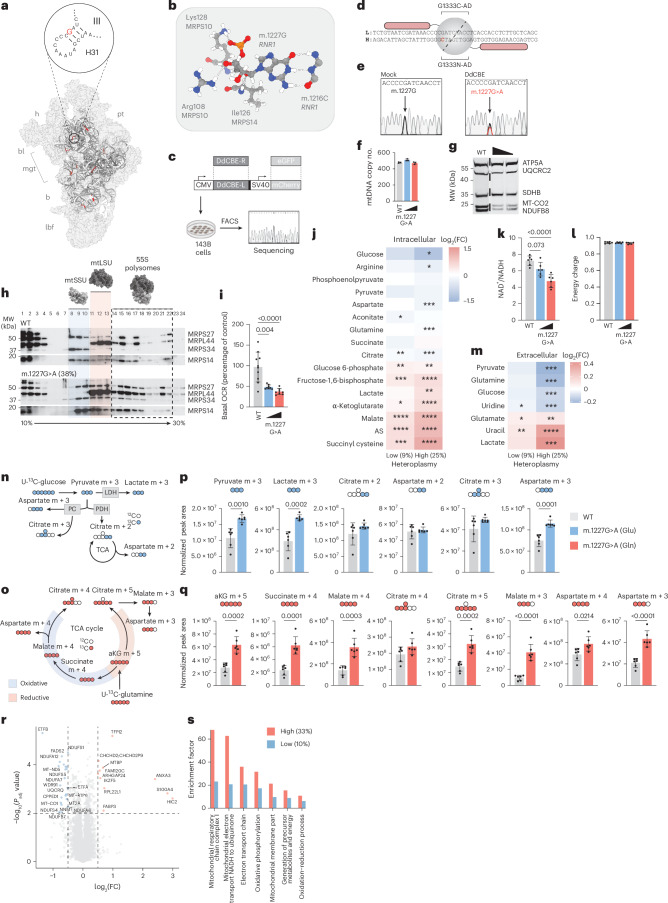
An exemplar rRNA hotspot, m.1227^G>A^, is functionally dominant. **a**, Location of m.1227^G^ within mtSSU. h, head; b, body; pt, platform; bl, beak lobe; lbf, lower body finger; mgt, mRNA gate-like feature. **b**, Local H-bonding (dotted white lines) of m.1227^G^ with components of mtSSU. **c**, Schematic of DdCBE screening workflow. FACS, fluorescence-activated cell sorting. **d**, Schematic of lead TALE-DdCBE pair for base editing of m.1227^G^. Targeted nucleotide is colored in red. Pink rods represent TALE binding domains. Gray sphere represents DdCBE catalytic domain. L, mtDNA light strand. H, mtDNA heavy strand. **e**, Representative Sanger sequencing trace of cells following treatment with control or m.1227^G>A^ DdCBE constructs. **f**, mtDNA copy number analysis. Three biological replicates are presented as individual datapoints. Measure of centrality is the mean. Error bars indicate 1 s.d. **g**, Western blot analysis of indicative respiratory chain subunits. This experiment was repeated several times and a representative result is presented. Sloped triangle indicates m.1227G>A 38–18% range. MW, molecular weight; WT, wild type. **h**, Sucrose gradient sedimentation analysis of mitochondrial ribosomes comparing WT and m.1227G>A (38%) cells. This experiment was performed twice; a representative result is presented. **i**, Basal oxygen consumption rate. This experiment was performed three times; a representative result is presented. Eight to ten separate wells are presented as individual datapoints. Measure of centrality is the mean. Error bars indicate 1 s.d. **j**, Heatmap of intracellular polar metabolite abundances. FC, fold change. AS, argininosuccinate. Six biological replicates are presented as individual datapoints across conditions. **k**, NAD^+^/NADH ratio of samples measured in **j**. Six biological replicates are presented as individual datapoints for each condition. Measure of centrality is the mean. Error bars indicate 1 s.d. **l**, Energy charge state analysis of samples measured in **j**. Six biological replicates are presented as individual datapoints for each condition. Measure of centrality is the mean. Error bars indicate 1 s.d. **m**, Heatmap of extracellular polar metabolite abundances. Six biological replicates are presented as individual datapoints for each condition. LDH, lactate dehydrogenase; PC, pyruvate carboxylase; PDH, pyruvate dehydrogenase. **n**, Schematic of U-^13^C-glucose carbon labeling. **o**, Schematic of U-^13^C-glutamine carbon labeling. **p**, Abundance of U-^13^C-glucose labeled metabolites, as indicated in **n**. Six biological replicates are presented as individual datapoints for each condition. Measure of centrality is the mean. Error bars indicate 1 s.d. **q**, Abundance of U-^13^C-glutamine labeled metabolites, as indicated in **o**. Six biological replicates are presented as individual datapoints for each condition. Measure of centrality is the mean. Error bars indicate 1 s.d. **r**, Volcano plot of protein abundances. Protein changes with a *P* value < 0.05 and log_2_(FC) > 0.5 are indicated. **s**, Pathway analyses of proteomic data. Three biological replicates are presented as one datapoint for each condition (**r**,**s**). Sloped triangles indicate low to high m.1227^G>A^ heteroplasmy as indicated in the text and legend. *P* values were determined using a one-way ANOVA test with Šídák (**j**), Fisher’s least significant difference test (**m**), Turkey’s multiple comparisons test (**k**,**l**), or two-tailed student’s t-test (**p**,**q**). For all panels: *P < 0.05; **P < 0.01; ***P < 0.001, ****P < 0.0001. Source data

**Extended Data Fig. 5:**
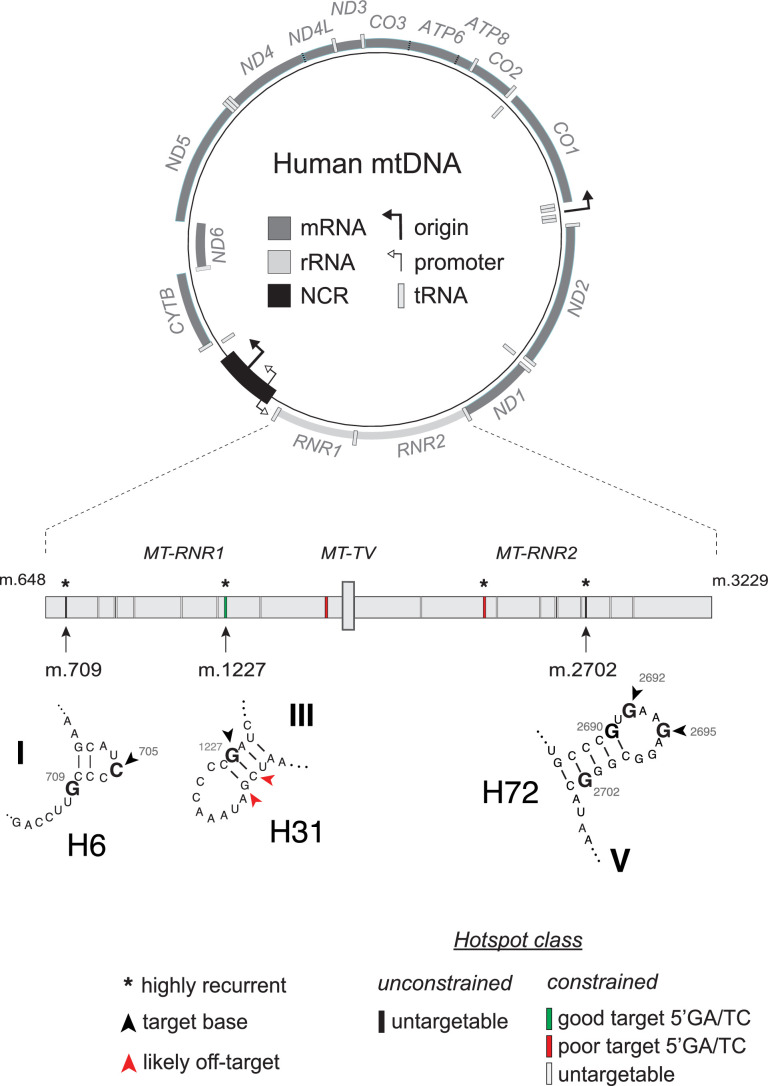
Schematic of mt-rRNA hotspots within human *MT-RNR1* & *MT-RNR2.* Individual recurrently mutated positions are indicated by rectangles within the expanded view of the region. The majority of hotspot mutations cannot be targeted using cytosine base editors, due to the neighbouring sequence context: key considerations 1) hotspot mutation must be C > T or G > A; 2) target base must be within 5’CT’3 or 5’GA’3 context; 3) target base must be in region amenable to TALE binding that conforms to usable linkers and editing window width. We selected m.709 and m.2702, both germline variant positions, due to exceptionally high recurrence. Direct targeting of the base to produce the observed mutation was not possible in either case, but editing to disrupt the structural feature within which they are contained was plausible. m.1227 G > A was the only hotspot in our initial analysis that was fully targetable by the above criteria.

**Extended Data Fig. 6:**
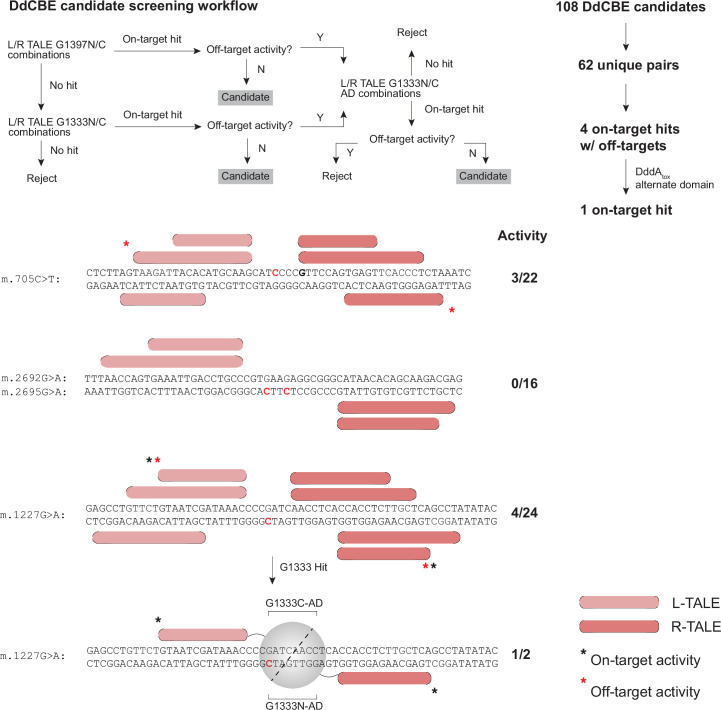
Scheme of iterative TALE screening for editing activity. In total, 108 DdCBE split pair candidates forming 62 unique DdCBE pairs using 1st generation DdCBE domains NC1397/1333 were assembled and screened in 143B cells as described (see Methods). The majority of these pairs demonstrated very low or no on-target editing. A minority demonstrated on-site off-target editing, and a single pair targeting m.1227 G > A demonstrated on-target editing with on-site off-target activity. This pair was taken forward and reassembled using a second generation DdCBE domain architecture as described, which maintained on target editing with greatly decreased off-target activity.

Given the atypical, unstudied nature of somatic mutations to mitochondrial ribosomal rRNAs, we sought to functionally characterize the effect of an rRNA hotspot allele. To do so, we examined the feasibility of using mtDNA-targeting DdCBE base editors to introduce the most commonly observed rRNA hotspots^22,23^. Three sites within *MT-RNR1* and *MT-RNR2* (m.709, m.1227 and m.2702) were selected for targeting on the basis of favorable sequence context and rate of recurrence within the genomic data (Extended Data Fig. 5). Of note, m.1227^G>A^ was the only hotspot with favorable sequence context to permit a direct edit. This nucleotide, located within the head domain of the mtSSU (Fig. 3a), forms several hydrogen bonds with residues in mS10 and mS14b protein subunits, in addition to base pairing with m.1216C as part of stem–loop H31, key to coordination of A-site tRNA within the mitoribosome^21^ (Fig. 3b). After screening of 62 unique DdCBE pairs against the three sites, m.1227^G>A^ alone demonstrated efficient TALE targeting of a DddA_tox_-mediated edit (Extended Data Fig. 6). Through iterative TALE design and DddA_tox_ domain screening in 143B cells, we identified a pairing capable of efficiently introducing m.1227^G>A^ (Fig. 3c–e), achieving up to ~40% m.1227^G>A^ heteroplasmy with a single transfection.

Cells harboring m.1227^G>A^ at heteroplasmic dosages between ~10% and ~40% demonstrated pronounced molecular phenotypes. Despite no observable change in total mtDNA copy number in cells bearing increasing levels of m.1227^G>A^ (Fig. 3f) (24–40% range), we observed clear, specific loss of protein expression for the complex I subunit NDUFB8 and the complex IV subunit MT-CO2 (18–38% range) (Fig. 3g). Using isokinetic sucrose gradient sedimentation analysis, we observed differential distribution and accumulation of both mtSSU and 55S polysomes in m.1227^G>A^ cells. Assembled mtSSU fractions (highlighted blue) demonstrated a distinct overlap with mtLSU fractions in m.1227^G>A^ cells (highlighted red), consistent with increased abundance of quality control factor-bound mtSSU and monosome intermediates. Increased abundance of polysomal fraction signal in m.1227^G>A^ cells coupled to decreased steady-state levels of NDUFB8 and MT-CO2 (Fig. 3h) further suggests that ribosome stalling events may underpin the loss of OXPHOS subunit expression^24,25^. In agreement with these findings, we observed that m.1227^G>A^ cells demonstrated a substantial depletion of basal oxygen consumption rate relative to wild-type cells (Fig. 3i) (24–40% range).

**Extended Data Fig. 7:**
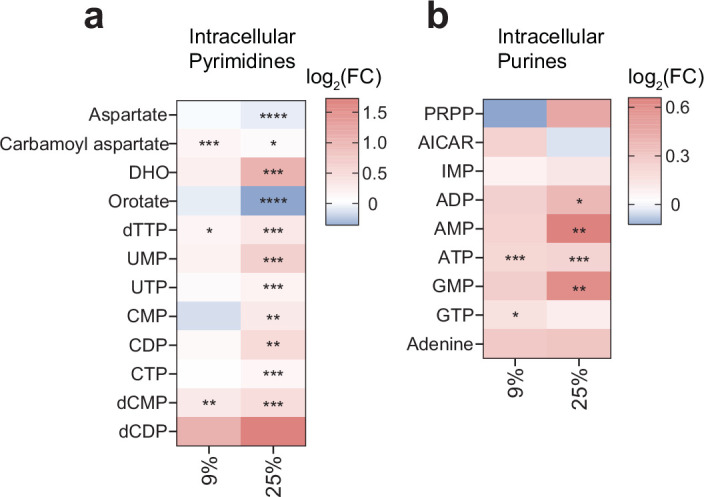
Altered nucleotide abundance in response to m.1227 G > A. **a**. Heatmap of detected pyrimidines, precursors and intermediates. **b**. Heatmap of detected purines, precursors and intermediates. FC, fold change. 6 biological replicates are presented as one datapoint for each condition. P values were determined using a one-way ANOVA test with Šídák’s multiple comparisons test.*p < 0.05; **p < 0.01; ***p < 0.001, **** p < 0.0001. Source data

To resolve the metabolic phenotype of m.1227^G>A^ more comprehensively, we assessed polar metabolite abundances using mass spectrometry (MS) at either low (9%) or high (25%) heteroplasmy. Relative to wild-type cells, m.1227^G>A^ cells demonstrated an increased abundance of redox-state-dependent metabolites such as lactate (m.1227G>A 25%, *P* = 0.0018), malate (m.1227G>A 9%, *P* < 0.0001; m.1227G>A 25%, *P* < 0.0001) and succinyl cysteine (m.1227G>A 9%, *P* = 0.0006; m.1227G>A 25%, *P* < 0.0001), alongside depleted glucose (m.1227G>A 25%, *P* = 0.0162) and pyruvate. In the context of elevated abundance for several glycolytic intermediates (glucose 6-phosphate (m.1227G>A 9%, *P* = 0.0058; m.1227G>A 25%, *P* = 0.0015), fructose 1,6-bisphosphate (m.1227G>A 9%, *P* = 0.0003; m.1227G>A 25%, *P* < 0.0001)), unchanged succinate and depleted aspartate levels (m.1227G>A 25%, *P* = 0.0002) (Fig. 3j), these changes indicate that the altered cellular NAD^+^:NADH ratio (m.1227G>A 25%, *P* < 0.0001) in mutant cells (Fig. 3k) could drive compensatory glycolytic flux to maintain cellular energetics (Fig. 3l) in response to diminished mitochondrial respiration, as has been observed previously in cells bearing engineered, cancer-associated mtDNA mutations at markedly higher heteroplasmy (60–80% range)^14^. Although oxygen consumption rate and steady-state profiling of intracellular metabolites from m.1227^G>A^ cells indicated mitochondrial dysfunction accompanied by depletion of tricarboxylic acid (TCA) cycle metabolites and key biosynthetic intermediates, consumption of glutamine (m.1227G>A 25%, *P* < 0.0001), glucose (m.1227G>A 25%, *P* = 0.0002), uridine (m.1227G>A 9%, *P* = 0.0324; m.1227G>A 25%, *P* < 0.0001) and pyruvate (m.1227G>A 25%, *P* < 0.0001) was greater in cells bearing the m.1227^G>A^ mutation for both low (9%) and high (25%) heteroplasmy cells (Fig. 3m). Increased nutrient consumption by m.1227^G>A^ cells co-occurred with greater secretion of lactate (m.1227G>A 9%, *P* = 0.002; m.1227 G > A 25%, *P* < 0.0001) and uracil (m.1227G>A 9%, *P* < 0.0001; m.1227G>A 25%, *P* < 0.0001) into the medium. Further, increased intracellular abundance of nucleotide precursors, pyrimidines and purines (Extended Data Fig. 7) within m.1227 ^G>A^ cells led us to speculate that m.1227^G>A^ may drive adaptive metabolic fluxes that support anabolism.

Using isotopically labeled U-^13^C-glucose and U-^13^C-glutamine (Fig. 3n,o), we assessed the metabolic fate of carbon supplied to m.1227^G>A^ (30%) cells and wild-type cells. Increases in both pyruvate m + 3 (*P* = 0.0010) and lactate m + 3 (*P* = 0.0002) from glucose in m.1227^G>A^ cells indicated greater glycolytic flux; however, pyruvate dehydrogenase-mediated supply of carbon to the TCA cycle remained stable between mutant and wild-type cells (citrate m + 2, aspartate m + 2). Anaplerotic supply of glucose-derived carbon to the TCA cycle via pyruvate carboxylase (citrate m + 3) remained stable; however, increased abundance of aspartate m + 3 was observed in mutant cells (*P* = 0.0001) (Fig. 3p). Increased utilization of glutamine (α-ketoglutarate m + 5, *P* = 0.0002) alongside increased labeling of intermediates derived from oxidative decarboxylation (succinate m + 4, *P* = 0.0001; malate m + 4, *P* = 0.0003) and reductive carboxylation of glutamine (citrate m + 5, *P* = 0.0002; malate m + 3, *P* < 0.0001) were observed in mutant cells, with significant increases in aspartate labeling from both pathways (aspartate m + 3, m + 4, *P* = 0.0214 and *P* < 0.0001, respectively) (Fig. 3q). These data indicate that depleted steady-state levels of key biosynthetic metabolites, such as aspartate and citrate (m.1227 G > A 9%, *P* = 0.0086; m.1227 G > A 25%, *P* = 0.0002) (Fig. 3j), are indicative of greater metabolic flux in m.1227^G>A^ cells rather than loss of TCA cycle activity. We carried out proteomic analysis of 143B m.1227 ^G>A^ and wild-type cells, where depletion in steady-state levels of respiratory chain subunits across all respiratory complexes bearing an mtDNA-encoded component, including MT-CO1, MT-ATP6, NDUFAF7, NDUFA12 and UQCRQ (Fig. 3r) was observed, with further proteins relevant to mitochondrial electron transport and metabolism, such as ETFA and ETFB, were also depleted in mutant cells (33% m.1227^G>A^). Gene ontology analyses of the proteomic data revealed a broad, heteroplasmy-dependent impact on the mitochondrial respiratory chain in cells bearing m.1227^G>A^ (Fig. 3s) (10–33% range).

### m.1227G>A induces heteroplasmy-dependent phenotypes

The limited functional data available for recurrent mtDNA alleles in cancer suggests that they are dosage-sensitive and functionally recessive^1,14^, that is, that the phenotype conferred by these mutations emerge at heteroplasmies over 50%, and that the mutations under strongest selection are driven to high heteroplasmy (or, in some cases, homoplasmy). In contrast to these observations, our multiparametric assessment of the proteomic and metabolic consequences of m.1227^G>A^ at modest heteroplasmy suggests that, even at dosages where most of the mtDNA pool remained wild-type, m.1227^G>A^ evoked a prominent metabolic phenotype, suggesting that this allele acted in a functionally dominant manner.

**Fig. 4:**
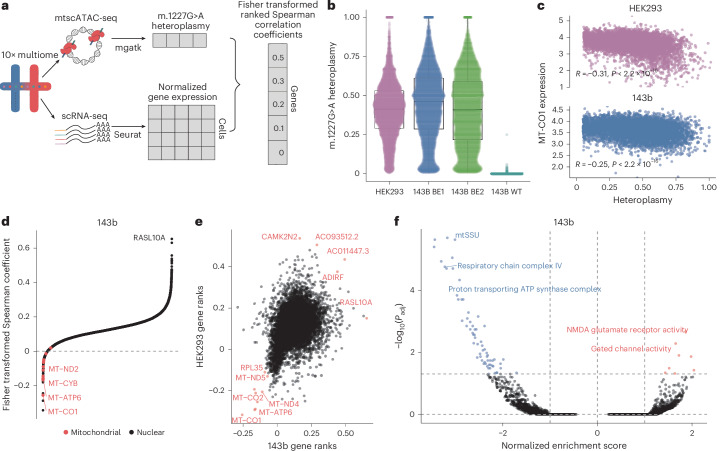
scRNA-seq demonstrates functional dominance of m.1227G>A rRNA hotspot mutation. **a**, Single-cell workflow. **b**, Heteroplasmy distribution of all single-cell libraries. Boxplots indicate median and interquartile range; error bars, S.E.M **c**, Correlation of *MT-CO1* expression with heteroplasmy across HEK293 (Spearman Correlation) and 143B BE1 library (Spearman Correlation). **d**, Sorted Fisher Z-transformed Spearman correlation ranks of heteroplasmy gene correlates from the 143B BE1 library. **e**, Association of HEK293 and 143B BE1 library gene ranks with selected genes annotated in red. **f**, GSEA volcano plot from 143B BE1 heteroplasmy gene correlates, negative normalized enrichment score (blue) represents downregulation with heteroplasmy whereas positive normalized enrichment score (red) represents upregulation with heteroplasmy. *P* values are adjusted using Benjamini–Hochberg correction.

**Extended Data Fig. 8:**
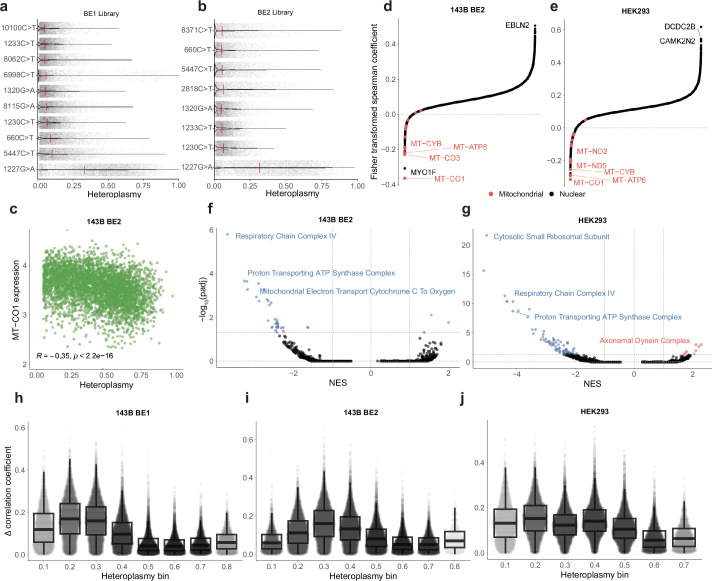
Functional dominance of the m.1227 G > A transcriptional phenotype. **a-b**. Heteroplasmy distribution of all somatic mutations in the 143B BE1 and BE2 libraries. The red line denotes the mean heteroplasmy. **c**. Correlation of heteroplasmy and *MT-CO1* gene expression in the 143B BE2 library (R = -0.35, p < 2.2e-6, Spearman Correlation). **d-e**. Fisher transformed gene expression - heteroplasmy ranked spearman correlation coefficients for the 143B BE2 library and the HEK293 library. Mitochondrial genes are coloured in red. **f-g**. GSEA using GO terms and the Fisher transformed gene list for both the 143B BE2 and HEK293 library. P-values are adjusted using Benjamin-Hochberg correction. **h-j**. Absolute values of change in the correlation coefficient across all genes within each 10% heteroplasmy bin across the HEK293 and 143B BE1 and BE2 libraries. Boxplots indicate median and interquartile range; error bars, S.E.M.

Although the above data are indicative, it is now well-appreciated that populations of cells with a defined bulk heteroplasmy are in fact constituted by cells harboring a wide distribution of heteroplasmies. Although such cell-to-cell heterogeneity in mtDNA heteroplasmy can confound bulk analyses, it can also be leveraged to study heteroplasmy-dependent phenotypes^26,27^. We employed a recently developed massively parallel sequencing protocol to concomitantly capture gene expression and accessible chromatin states at single-cell resolution in two replicate libraries derived from m.1227^G>A^ (‘BE1’ and ‘BE2’) 143B libraries, in addition to a third HEK293 library (Fig. 4a). We successfully captured 16,685 cells across the two mutant 143B libraries and 8,122 in the HEK293 library with high coverage (>25×) of the m.1227 locus, enabling us to calculate the heteroplasmy of m.1227^G>A^ with high precision. The mtDNA of individual cells was genotyped using the ATAC component of each multiome library, which revealed low rates of additional somatic mutations in both 143B libraries (Extended Data Fig. 8a,b), including evidence of off-target editing at two positions proximal to m.1227 (m.1230, m.1233, mean single-cell heteroplasmy 6.06% and 5.17%, respectively), as well as an additional heteroplasmic variant (m.9369) endogenous to 143B cells present at a mean 29.4% heteroplasmy. Similarly, in the HEK293 cells, we identified the potential off-target m.1230 mutation (mean heteroplasmy of 5.6%). Across all cells, the median heteroplasmy of the mutant m.1227 allele was 33% (143B BE1), 31% (143B BE2) and 39% (HEK293). Heteroplasmy distributions in all three libraries were strongly skewed towards zero, with 31%, 30% and 15% (for 143B BE1, 143B BE2 and HEK293, respectively) of cells having a heteroplasmy <10% (Fig. 4b)—an observation that was robust to the removal of cells with off-target edits at m.1230 and m.1233 for both 143B and HEK293 libraries.

To model quantitatively the dependence of gene expression on m.1227^G>A^ heteroplasmic dosage, we calculated the nonparametric (Spearman) correlation between the expression of each gene and m.1227^G>A^ heteroplasmy across 143B and HEK293 cells (Methods). A small number of genes, concentrated in genes encoded in mtDNA itself, demonstrated strong negative association with heteroplasmy (Fig. 4d,e and Extended Data Fig. 8d,e). For example, *MT-CO1* demonstrated a progressive decrease in gene expression as heteroplasmy increased (Fig. 4c,d and Extended Data Fig. 8c)—an effect that was apparent even at heteroplasmies well below 50%. This effect was unexpected, as both bulk and single-cell sequencing data have suggested that pathogenic truncating mutations elicit a compensatory increase in mitochondrial gene expression that is positively associated with heteroplasmy^3,28^. To understand the pathways associated with heteroplasmic adaptation, we carried out gene set enrichment analysis (GSEA) on correlation coefficients derived from the gene expression/heteroplasmy analysis above (Methods). This revealed several distinct subsets of genes that were consistently downregulated across sequencing libraries at high heteroplasmy levels, including nuclear-DNA-encoded genes associated with oxidative phosphorylation, as well as genes encoding both cytosolic and mitochondrial ribosomal subunits (Fig. 4f and Extended Data Fig. 8f,g). Finally, we hypothesized that if m.1227^G>A^ acted in a functionally dominant manner, it would demonstrate threshold effects at characteristically lower heteroplasmy levels. Consistent with this, unsupervised threshold analysis (Methods) revealed the greatest change in heteroplasmy-gene correlations at heteroplasmic dosages of 0.2–0.3 across both 143B and HEK293 cells. These data, together with our population-scale data indicating that m.1227 is under strong purifying selection in the germline, indicates that m.1227^G>A^ is a disruptive, functionally dominant mtDNA mutation that elicits effects on mitochondrial respiration, metabolism and cellular translation.

## Discussion

Although the pathological and clinical landscape of germline mtDNA mutations is well established, the impact of prevalent somatic mtDNA mutations, present in approximately half of all tumors, remains a point of contention. Past work has identified recurrent truncating mutations in mtDNA-encoded genes in tumors, which have been shown to modify tumor metabolism, immune microenvironments and response to therapy^1,14^. In the germline more broadly, and also in the context of somatic truncating mutations in tumors, phenotypes are observed in a fashion that is consistent with functionally recessive alleles, that is, heteroplasmic loads greater than 50% are required for phenotypes to manifest.

Leveraging the most comprehensive tumor mtDNA sequencing cohort to date, we have developed a high-resolution overview of previously unidentified mtDNA hotspot mutations in both coding and non-coding genes, allowing us to identify and functionally validate recurrent mutation of the mt-rRNA in conserved, germline-constrained positions that arise in ~4% of tumors across all tumor types. Whereas somatic hotspots tend to affect positions under purifying selection in the germline, the mtDNA germline has been acted on by selection at population-scale for thousands of years, whereas tumor mtDNA affects a tissue-lineage-restricted subset of cells often at low oxygen tension and with plentiful substrates, limiting the conclusions of a comparison between the two contexts. Through mtDNA base editing, we introduced the m.1227^G>A^ mutation into human cancer cells, providing clear evidence of heteroplasmy-dependent mitochondrial dysfunction owing to mutation at this conserved position.

Of particular interest is the heteroplasmy at which such phenotypes manifest; typically germline mutation of mtDNA does not cause mitochondrial disease or detectable biochemical dysfunction at heteroplasmies below ~60%—a phenomenon known as the threshold effect, which positions mtDNA mutation as a functionally recessive class of genetic defect^11^. Mutations of mt-rRNA are not a known cause of hereditary mitochondrial disease, and given the extreme impact of mutations in rRNA it is logical that phenotypes would be manifested at low heteroplasmies, as has been seen previously in experimentally induced chloramphenicol resistance-associated mt-rRNA variants^29,30^. Although we have been able to functionally characterize an exemplar of the mt-rRNA hotspot class, modeling of further variants was not possible owing to the technical difficulty of targeting precision edits within the defined nucleotide sequence context of disease-associated variants. However, given the shared patterns of genomic conservation, heteroplasmic dosages and structural features of the mt-rRNA hotspots we describe, we think it probable that the phenotype of m.1227^G>A^ will be generalizable to others in this class. Our data suggest that these variants could be considered the first recurrent, functionally dominant mutations of mtDNA, with evidence of selective pressure and deleterious impact well below the threshold seen for hereditary mtDNA disease variants.

The impact of m.1227^G>A^ on the mitoribosome seems to be broad, potentially ranging from defective assembly of mtSSU and monosome formation through to stalling of assembled, translating mitoribosomes. Although it seems m.1227^G>A^ does not entirely preclude mtSSU assembly, it is tempting to speculate that the dominant negative characteristics of m.1227^G>A^ could be explained by formation of subfunctional monosomes, whereas m.1227^G>A^-containing mtSSU is bound to wild-type mtLSU, limiting availability of mtLSU to form functional monosomes with wild-type mtSSU. m.1227^G>A^ monosome stalling on mature mt-mRNAs, preventing translation of these by functional monosomes, may also explain the disproportionate phenotypic consequences of this mutation at low heteroplasmy.

Beyond clear differences in phenotypic mutant allele dosage threshold compared to previously observed disease-associated mtDNA variants, the metabolic phenotypes exhibited by m.1227^G>A^ further revealed a point of divergence between mutations in rRNA and protein-coding regions. Whereas loss of specific electron transport chain complex activity due to mutation in protein-coding genes often results in loss of OXPHOS and TCA cycle activity, cells bearing m.1227^G>A^ demonstrated distinctive features of an alternate metabolic adaptation. Taken together, greater consumption of nutrients coupled to enhanced anaplerotic labeling of TCA cycle derived metabolites despite depletion of the total pool size of the same metabolites (for example, citrate, aspartate) is consistent with greater catabolic and anabolic capacity of cells bearing m.1227^G>A^. Given the observed changes in mitochondrial protein content of mutant cells relative to wild type are largely restricted to the electron transport chain complexes, and that mtDNA copy number does not vary between wild type and mutant states, a form of metabolic plasticity seems to be established by mutation-dependent disequilibrium between TCA cycle fluxes and a lower overall OXPHOS capacity. Low heteroplasmy hotspot mutation in MT-RNR genes may be similar to high heteroplasmy mutation of mitochondrial tRNAs in this regard, where functionally wild-type OXPHOS complexes are assembled at a dramatically decreased steady-state level, manifesting in a profoundly perturbed cellular redox state that can be maintained only through increased metabolic activity. Mutation of mtDNA in tumors is currently thought to be an early event^31^. As such, it is tempting to speculate that hotspot mutations in mt-rRNA may support cell proliferation and induce nucleotide imbalances that lead to genome instability^32–34^.

We observed an intriguing interaction between the mitochondrial and cytosolic translation processes, as has been seen previously^35,36^, where notable mitochondrial translation defects influence the nature of core cellular processes, probably beyond the direct metabolic effects of mitochondrial dysfunction. A similar process, where regulation of cytosolic translation occurs in an mtDNA mutation-dependent manner has been described recently in oocytes, where strong purifying selection of deleterious mtDNA variants is well established^37^. Influences on cytosolic translation may therefore explain both the functionally dominant nature of mt-rRNA mutation, and the strong negative selection at the level of heteroplasmy that seems to operate on germline-constrained hotspot mutations like m.1227^G>A^.

Mutation of nonredundant elements in the central dogma are not observed in biology, and are broadly considered to be incompatible with life. Therefore, somatic gain of function mutations in mitochondrial rRNA represent both a new class of cancer-associated mutation and a new class of disease-relevant, functionally dominant mtDNA mutation that is observed only through the biologically permissive lens of cancer. More conventional pathogenic mtDNA variants, demonstrating functionally recessive characteristics, are known to meaningfully impact tumor phenotypes and therapeutic susceptibility^14,38^. The existence of such exotic and extreme genetic variants in mitochondrial ribosomal RNA, implicitly linked to profound metabolic remodeling in tumors, compels an appreciation for the broader functional and therapeutic relevance of mitochondrial DNA mutations in cancer, while revealing a common disease state where the established rules of mitochondrial genetics no longer apply.

## Methods

### Ethics

This study uses publicly accessible, anonymized patient sequencing data from several consortia (GEL, TCGA, PCAWG, Helix). This study did not directly recruit or collect data from human participants and does not make use of animal models and therefore was not subject to specific ethical approvals.

### Genomics England cohort

Primary tumors with matched-normal sequencing data were obtained from the Genomics England Consortium, as part of the 100,000 Genomes project, to yield a total of 14,106 unique genomes (v.18 release). Data for the 100,000 Genomes project is available directly from Genomics England^15^. Genomics England has obtained written informed consent from all participants. If a patient’s primary tumor was profiled more than once, only the sample with the highest tumor purity was retained. In total, the dataset was comprised of 57% female and 43% male patients, with a median age of 72 years. Alignment of bulk WGS data to the human reference genome was performed as part of the 100,000 Genomes project, and therefore, reads mapping to the mitochondrial genome were extracted directly from the preprocessed BAM file to perform variant calling. As WGS yields high coverage across the entirety of the mitochondrial genome, all samples were retained for subsequent analysis.

### Somatic mitochondrial mutation calling

mtDNA variants were called in the WGS data using our previously established bulk-variant-calling pipeline^1,28^. Briefly, variant calls from Mutect2 using mitochondrial mode were intersected with the variant calls from our inhouse variant caller, which is based on the SAMtools mpileup utility and takes in tumor and matched normal for somatic variant calls. The resulting somatic variants in hypermutated blacklisted regions (513–525 and 3105–3109) were filtered out. Next, variants with heteroplasmy of >5% in the tumor and <1% in the matched normal and supported by at least two reads in both the tumor’s forward and reverse strands were retained. As previous work called mtDNA variants at lower hetereroplasmies (1%), we called mtDNA somatic variants with this more sensitive threshold. However, due to the presence of artifacts, marked by the noncanonical enrichment of T>G mutations (Extended Data Fig. 1b), we concluded that the 5% heteroplasmy threshold offers a more specific identification of somatic mtDNA mutations.

### Identification and analysis of hotspot mutations

mtDNA positions with statistically recurrent SNVs were identified by comparing the observed proportion of mutations at an individual position (out of the total number of mutations acquired in its gene) with the rate of mutations at the position as expected by chance with a one-sided binomial test^1^. Control regions and other blacklisted regions were removed. A Bernoulli trial was used to model the probability of a SNV at each position of a gene (*P*_pos,gene_), where the likelihood of a mutation arising at a given position is defined by its mutability (*μ*) relative to the mutability of all other bases in the gene: *P*_pos,gene_ = *μ*_pos_/*μ*_gene_. More precisely, to calculate the probability of a mutation at any given position, we considered both the mutability of that position’s trinucleotide context (*μ*_pos_) and the mutability of the gene in which it lies (*μ*_gene_). Consistent with previous work, *μ*_pos_ was determined by tabulating the mutation frequency of each of the 64 possible trinucleotide contexts and dividing by the total number of SNV mutations observed across the entire genome. Then, *μ*_gene_ was taken as the sum of all *μ*_pos_ with nonzero mutation rates in that gene. The likelihood of a mutation in a gene arising at a given position by chance was therefore *P*_pos,gene_ = *μ*_pos_/*μ*_gene_. Positions are tested for statistically enriched mutations by comparing its observed number of mutations out of the total mutations in the gene with this binomial parameter, using a one-sided binomial test. *Q*-values were generated across all mutant alleles post Benjamin–Hochberg correction. To validate hotspot calls, we compared the incidence of mutations in the GEL cohort to two additional cohorts, PCAWG and TCGA, which our group had analyzed previously^1^ (Extended Data Fig. 3a,b.).

### Structural annotation of rRNA genes

Each position along the ribosomal gene secondary structure was annotated as either Watson–Crick base pairing, loop-or-other, or non-Watson–Crick as previously defined^39^.

### Comparisons to germline variation

We classified each position in mtDNA according to its tolerance for a germline homoplasmy in the Helix database of mitochondrial germline variation, labeling positions with a population allele frequency of <0.00001% (fewer than 2 of 196,554 people harboring the allele) as ‘germline constrained.’ Common pathogenic germline polymorphisms and rates of evolutionary conservation were retrieved from the MITOMAP mitochondrial database^19^. Incidences of all homoplasmic polymorphisms were identified from the HelixMTDb^20^. Certain positions of the mitochondrial genome are not reported in the HelixMTDb, indicating that these positions were never reported to have a homoplasmic polymorphism. Therefore, for every position in the mitochondrial genome, from 1 to 16569, we set homoplasmic mutation counts to zero for such loci.

### Mitochondrial and nuclear mutation co-occurrence analysis

Precomputed somatic nuclear mutation calls from the GEL cohort were filtered to include the 468 genes in the targeted MSK-IMPACT sequencing panel. We then tested co-occurrence of these known nuclear driver gene mutations, within each cancer type, at five levels of mtDNA SNV alterations: (1) any mtDNA mutation, (2) mtDNA truncating mutations, (3) mtDNA hotspot mutation, (4) any mt-rRNA mutation and (5) mt-rRNA hotspot mutation. At each level of mtDNA alteration, co-occurrence was assessed for nuclear genes that were mutated in at least 5% of patients within that cancer type using a chi-squared test. *P* values were adjusted using Bonferroni–Hochberg correction.

### Tissue-enrichment analysis

To test for tissue enrichment or depletion of mt-rRNA hotspot mutations, we assessed systematically whether any given tissue had a greater than expected rate of mt-rRNA hotspot mutations. To do so, we applied a two-sided binomial test where the number of total trials, *n* = number of patients in the tissue; the number of successes, *x* = number of observed mt-rRNA hotspot mutations, and the probability of success *P* = number of patients with an rRNA hotspot mutation per total number of patients (Supplementary Table 4).

### Maintenance of cell lines and culture

Osteosarcoma cells (143B) were maintained in 4.5 g l^−1^ d-glucose DMEM medium supplemented with 10% fetal bovine serum (FBS), L-glutamine, and penicillin and streptomycin (Pen/Strep, Life Technologies). Cells were transfected and sorted as published previously^14^. After sorting cells were maintained in 4.5 g l^−1^ d-glucose DMEM medium supplemented with 20% FBS, 100 μg ml^−1^ Uridine (Sigma), L-glutamine and Pen/Strep.

### DdCBE m.1227G>A construct design

Candidate TALE sequences were cloned into either pcDNA3.1-mCherry (1227R1) or pTracer CMV/Bsd2-GFP (1227L1) expressing plasmids^14^ using *Eco*RI and *Bam*HI. Initial library screening using first generation DddA_tox_ G1397/G1333 domains failed to yield pairs with efficient onsite activity. Further screening of the TALE library fused to the DddA11 domain variant^23^ in G1333 split orientation yielded efficient onsite activity.

### DNA isolation and determination of m.1227 heteroplasmy

DNA from cell pellets was isolated using DNeasy Blood and Tissue Kit (Qiagen) as per the manufacturer’s instructions. Phusion DNA polymerase PCR (Thermo Scientific) reaction was performed using primers with a forward primer containing a T7 primer binding site to allow for subsequent T7 primer Sanger sequencing. Before sequence submission, samples were purified using a QIAquick PCR purification kit (Qiagen). Heteroplasmy was determined by comparing peak heights at m.1227 for either the G or the A nucleotides. Note that pyrosequencing could not be used due to the presence of a nuclear mitochondrial pseudogene.

### Immunoblotting

Cells were washed twice with ice-cold PBS and lysed in ice-cold RIPA buffer (Life Technologies) supplemented with cOmplete Mini Tablets and cOmplete Mini Protease Inhibitor Tablets (Roche) for 10 min on ice and centrifuged at 14,000*g* for 10 min at 4 °C. Total protein concentration in the supernatant was determined by DC Protein Assay (Bio-Rad Laboratories). Equal amounts of proteins were resolved on precast SDS–PAGE 4–12% Bis-Tris Bolt gels (Life Technologies). Proteins were transferred to nitrocellulose using a Mini Trans-Bolt Cell (Bio-Rad Laboratories). Membranes were incubated overnight with primary antibodies, and secondary antibodies were incubated at 1:5,000 dilution for 1 h. Immunoblots were analyzed using an Odyssey CLx imager and Image Studio Lite software (Li-COR Biosciences). Antibodies used were Total OXPHOS Human WB antibody cocktail (1:1,000; ab110411, Abcam) and IRDye 800CW Donkey anti-Mouse IgG Secondary Antibody (1:5,000; 926-32212, Licor).

### Determination of mtDNA copy number

Cell DNA samples were analyzed in triplicate in 20 μl reactions containing 1 ng of genomic DNA, 100 nM of each primer, 10 µl of QX200 ddPCR EvaGreen Supermix and water using the QX200 Droplet Digital PCR System (Bio-Rad Laboratories) with the primer annealing temperature set at 54 °C.

### Sucrose gradient sedimentation analysis

Cells were lysed with 1% n-dodecyl β-D-maltoside lysis buffer (50 mM Tris, pH 7.2, 10 mM Mg(CH_3_COO)_2_, 40 mM NH_4_Cl, 100 mM KCl, 1% n-dodecyl β-D-maltoside, 1 mM phenylmethylsulfonyl fluoride, 1.24 mM chloramphenicol and 1 mM ATP) by incubating the samples for 20 min on ice followed by centrifugation at 20,000*g* for 20 min at 4 °C. Protein concentration in the supernatant was determined by Bradford assay and 1 mg of protein from each sample was loaded on top of a 7.5 ml linear 10–30% sucrose gradient (50 mM Tris, pH 7.2, 10 mM Mg(CH_3_COO)_2_, 40 mM NH_4_Cl, 100 mM KCl, 1 mM phenylmethylsulfonyl fluoride and 1 mM ATP). Samples were centrifuged for 15 h at 4 °C and 74,400*g*. The gradients were divided into 24 fractions of equal volume from top to bottom, precipitated with trichloroacetic acid and subsequently used for immunoblotting.

### Oxygen consumption rate measurements

The Seahorse XF Cell Mito Stress Test (Agilent) was performed as per the manufacturer’s instructions. In brief, cells were plated at 20,000 cells per well in a XFe96 plate (Agilent) 1 day before the analysis in regular growth medium. The next day cells were equilibrated for 1 h at 37 °C in bicarbonate-free 150 µl Seahorse XF medium supplemented with 1% FBS, 25 mM glucose, 1 mM sodium pyruvate and 2 mM glutamine. Oxygen consumption rate and extracellular acidification rate were measured three times every 9 min using a Xfe96 Analyzer (Seahorse Bioscience) at a baseline and after addition of each drug. To assess the mitochondrial respiratory ability, oligomycin (1 μM), carbonyl cyanide m-chlorophenyl hydrazone (1 μM), rotenone (1 μM) and antimycin A (1 μM) (all from Sigma) were injected subsequently. Afterwards, protein concentration was measured using the DC assay for normalization.

### In vitro metabolomics

Cells were seeded at 30,000 cells per well in sextuplicates in a 12-well plate. The following day, cells were replenished with fresh medium for steady-state experiments or medium containing labeled isotopes. To extract extracellular metabolites, medium from each well was centrifuged at 300*g* for 5 min and 20 µl supernatant was added to 980 µl of extraction buffer. To extract intracellular metabolites, cells were washed twice with ice-cold PBS and 200 µl of extraction buffer (50/30/20, v/v/v, methanol/acetonitrile/water) was added to each well and incubated for 5 min at 4 °C. All samples were centrifuged at 16,000*g* for 10 min at 4 °C and the supernatant was transferred to liquid chromatography (LC)–MS (LC–MS) glass vials and stored at −80 °C until run on the mass spectrometer. Compound peak areas were normalized using the total protein concentrations per well as quantified using a Lowry protein assay.

For U-^13^C-glucose isotope tracing experiments, medium was prepared as follows: DMEM, no glucose (Life Technologies) supplemented with 0.11 g l^−1^ sodium pyruvate, 2 mM l-glutamine, 20% FBS, 100 µg m^−1^ uridine and 25 mM glucose isotope (Cambridge Isotopes). For isotope tracing experiments using U-^13^C-glutamine, DMEM containing 4.5 g l^−1^ d-glucose and 0.11 g l^−1^ sodium pyruvate was supplemented with 20% FBS, 100 µg ml^−1^ uridine and 4 mM glutamine isotope (Cambridge Isotopes).

### Data analysis for metabolomics

Metabolite extracts were analyzed using a ZIC-pHILIC column (SeQuant; 150 mm × 2.1 mm, 5 µm; Merck) and ZIC-pHILIC guard column (SeQuant; 20 mm × 2.1 mm; Merck) coupled to a Vanquish HPLC system (Thermo Fisher Scientific). A gradient program was employed, using 20 mM ammonium carbonate (pH 9.2, 0.1 % v/v ammonia, 5 µM InfinityLab deactivator (Agilent)) as mobile phase A and 100% acetonitrile as mobile phase B. Elution started at 20% A (2 min), followed by a linear increase to 80% A for 15 min and a final re-equilibration step to 20% A. Column oven was set to 45 °C and flow rate to 200 µl min^−1^. A Q Exactive Plus Orbitrap mass spectrometer (Thermo Fisher Scientific) equipped with electrospray ionization was used. Instrument was used in polarity switching mode, resolution of 70,000 at 200 *m*/*z*, mass range 75–1,000 *m*/*z*, automatic gain control target of 1 × 10^6^ and maximal injection time of 250 ms. Metabolite identification was performed by matching accurate mass and retention time of observed peaks to an inhouse library generated using metabolite standards (mass tolerance of 5 ppm and retention time tolerance of 0.5 min). To account for batch-effects, transcripts per kilobase million normalization was performed across each replicate as such: (Abundance_*i*_/sum(Abundance_*i* = 1 → *n*_) × 1 × 10^−6^, where *i* represents a metabolite and *n* is the total number of measured metabolites. Then, the geometric mean across replicates for each metabolite was used to calculate the fold change with respect to the wild-type and t.test() was performed to determine differentially up or downregulated metabolites.

### Sample preparation for proteomic MS analysis

Cells were washed twice with ice-cold PBS and scraped in lysis buffer (2% SDS in 100 mM Tris-HCl pH 8). Lysates were sonicated four times for 5 s and centrifuged at 16,000*g* for 5 min at room temperature. Detergent compatible (DC) protein assay was performed on the supernatants. Equal protein from the different samples were reduced with 10 mM dithiothreitol and subsequently alkylated in the dark with 55 mM iodoacetamide, both reactions were carried out at room temperature. Alkylated proteins were precipitated adding four volumes of acetone, samples were kept at −20 °C overnight. Washed pellets were reconstituted in 50 µl of HEPES buffer 200 mM and digested first with Endoproteinase Lys-C (ratio 1:33 enzyme:lysate) for 1 h, followed by trypsin overnight (ratio 1:33 enzyme:lysate). The digested peptides from each experiment, and a pool sample, were labeled differentially using TMT10-plex reagent (Thermo Scientific). Each sample was labeled with 0.1 mg of tandem mass tag (TMT) reagent dissolved in 50 μl of 100% anhydrous acetonitrile. The reaction was carried out at room temperature for 2 h. Fully labeled samples were mixed in equal amounts and desalted using a 50 mg Sep Pak C18 reverse-phase solid-phase extraction cartridges (Waters).

### High pH peptide fractionation

TMT-labeled peptides were fractionated using high pH reverse-phase chromatography on a C18 column (150 × 2.1-mm internal diameter Kinetex EVO (5 μm, 100 Å)) on a HPLC system (LC 1260 Infinity II, Agilent). A two-step gradient was applied, from 1% to 28% B in 42 min, then from 28% to 46% B in 13 min to obtain a total of 21 fractions for MS analysis.

### Ultra high performance LC–MS/MS analysis

Fractionated peptides were separated by nanoscale C18 reverse-phase LC using an EASY-nLC II 1200 (Thermo Scientific) coupled to an Orbitrap Q Exactive HF mass spectrometer (Thermo Scientific)^14^. Elution was carried out using a binary gradient with buffer A (water) and B (80% acetonitrile), both containing 0.1% formic acid. Samples were loaded with 6 µl of buffer A into a 50-cm fused silica emitter (New Objective) packed inhouse with ReproSil-Pur C18-AQ, 1.9 μm resin (Dr Maisch). Packed emitter was kept at 50 °C by means of a column oven (Sonation) integrated into the nanoelectrospray ion source (Thermo Scientific). Peptides were eluted at a flow rate of 300 nl min^−1^ using different gradients optimized for three sets of fractions: 1–7, 8–15 and 16–21 (ref. ^40^). Each fraction was acquired for a duration of 190 min. Eluting peptides were electrosprayed into the mass spectrometer using a nanoelectrospray ion source (Thermo Scientific). An active background ion reduction device (ESI Source Solutions) was used to decrease air contaminants signal level. The Xcalibur software (Thermo Scientific) was used for data acquisition. A full scan over mass range of 375–1,400 *m*/*z* was acquired at 60,000 resolution at 200 *m*/*z*, with a target value of 3 × 10^6^ ions for a maximum injection time of 20 ms. Higher energy collisional dissociation fragmentation was performed on the 15 most intense ions selected within an isolation window of 0.8 *m*/*z*, for a maximum injection time of 75 ms or a target value of 100,000 ions. Peptide fragments were analyzed in the Orbitrap at 45,000 resolution.

### Analysis of proteomic data

The MS Raw data were processed with MaxQuant software^41^ v.1.6.14.0 and searched with Andromeda search engine^42^, querying SwissProt^43^ Homo sapiens (42,438 entries). First and main searches were performed with precursor mass tolerances of 20 ppm and 4.5 ppm, respectively, and MS/MS tolerance of 20 ppm. The minimum peptide length was set to six amino acids and specificity for trypsin cleavage was required, allowing up to two missed cleavage sites. MaxQuant was set to quantify on ‘Reporter ion MS2,’ and TMT10 plex was chose as Isobaric label. Interference between TMT channels were corrected by MaxQuant using the correction factors provided by the manufacturer. The ‘Filter by PIF’ option was activated and a ‘Reporter ion tolerance’ of 0.003 Da was used. Modification by Iodoacetamide on cysteine residues (Carbamidomethylation) were specified as fixed, whereas methionine oxidation and N-terminal acetylation were allowed as variable modifications. The peptide, protein and site false discovery rate (FDR) was set to 1%. The proteinGroups.txt file from MaxQuant output was used for protein quantitation analysis using Perseus software v.1.6.15.0^41^. The ‘Reverse,’ ‘Potential Contaminants’ and ‘Only identified by site’ protein, as specified in MaxQuant, were removed, as well as protein groups identified with no unique peptides. Only proteins robustly quantified in all replicates in at least one group, were allowed in the list of quantified proteins. Significantly enriched proteins were selected using a permutation-based Student’s *t*-test or ANOVA with FDR set at 5%.

### Single-cell multiome

Single-cell multiome assay for transposase-accessible chromatin using sequencing (ATAC-seq) + Gene Expression was performed with 10x Genomics system using Chromium Next GEM Single Cell Multiome Reagent Kit A (cat. no. 1000282) and ATAC Kit A (cat. no. 1000280). We followed Chromium Next GEM Single Cell Multiome ATAC + Gene Expression Reagent Kits User Guide with modifications to the single-cell suspension with 0.3% NP-40 concentration and 3’ of lysis for optimal mtDNA. These buffer modifications are required to capture mtDNA reads with the multiome kit as originally described in ref. ^44^ and refined (ref. ^45^) for optimal mtDNA yields. Libraries were sequenced on Illumina NovaSeq X+ platform with standard read configurations for gene expression (R1, 28 cycles; R2, 90 cycles) and the mtDNA-enriched ATAC arm (R1, 50 cycles; R2, 50 cycles; i5, 24 cycles).

### Analysis of single-cell RNA sequencing and ATAC-seq and calculation of heteroplasmy

Raw single-cell RNA sequencing (scRNA-seq) and ATAC-seq reads were aligned to the human genome GRCh38 using CellRanger-Arc (v.2.0.2). Genotyping and heteroplasmy calculation of m.1227G>A was performed using mgatk v.0.7.0 with a masked reference genome^46^. Gene expression data was processed using Seurat (v.4.3.0) in R (v.4.4.1). Cells with fewer than 1,000 features and greater than 7,000 features and with greater than 5% mitochondrial gene expression were filtered out. Furthermore, only cells that had greater than 25 reads and fewer than 250 reads at position m.1227 were retained to ensure confidence in heteroplasmy estimation. Gene expression data were subsequently normalized and scaled before being run through the RunUMAP and FindClusters functions with resolution of 0.1. Heteroplasmy calculation was conducted as follows:

$${\rm{H}}{\rm{e}}{\rm{t}}{\rm{e}}{\rm{r}}{\rm{o}}{\rm{p}}{\rm{l}}{\rm{a}}{\rm{s}}{\rm{m}}{\rm{y}}=\frac{({\rm{N}}{\rm{u}}{\rm{m}}{\rm{b}}{\rm{e}}{\rm{r}}\,{\rm{o}}{\rm{f}}\,{\rm{a}}{\rm{l}}{\rm{t}}{\rm{e}}{\rm{r}}{\rm{n}}{\rm{a}}{\rm{t}}{\rm{e}}\,{\rm{r}}{\rm{e}}{\rm{a}}{\rm{d}}{\rm{s}})}{({\rm{N}}{\rm{u}}{\rm{m}}{\rm{b}}{\rm{e}}{\rm{r}}\,{\rm{o}}{\rm{f}}\,{\rm{a}}{\rm{l}}{\rm{t}}{\rm{e}}{\rm{r}}{\rm{n}}{\rm{a}}{\rm{t}}{\rm{e}}\,{\rm{r}}{\rm{e}}{\rm{a}}{\rm{d}}{\rm{s}}+{\rm{N}}{\rm{u}}{\rm{m}}{\rm{b}}{\rm{e}}{\rm{r}}\,{\rm{o}}{\rm{f}}\,{\rm{r}}{\rm{e}}{\rm{f}}{\rm{e}}{\rm{r}}{\rm{e}}{\rm{n}}{\rm{c}}{\rm{e}}\,{\rm{r}}{\rm{e}}{\rm{a}}{\rm{d}}{\rm{s}})}$$

### Correlation of gene expression and heteroplasmy

To determine which genes were associated most strongly with m.1227G>A heteroplasmy, we calculated the Spearman correlation between each gene’s expression and heteroplasmy. We filter out cells that do not express the gene, and only test the association with genes where more than 50 cells have nonzero gene expression. The coefficient correlations were subsequently Fisher *Z*-transformed by taking there hyberbolic inverse tangent and sorted to produce a ranked gene list. A secondary approach we used to challenge the robustness of our results, and to control for cell heterogeneity, was a linear regression model controlling for m.9369T>C, m.1230C>T and m.1233C>T heteroplasmies in the 143B cell line: lm(gene_expression ~ m.1227_heteroplasmy + m.9369_heteroplasmy + m.1230_heteroplasmy + m.1233_heteroplasmy). This model was run on all genes, and regression coefficients and *P* values associated with the m.1227 variant were subsequently extracted and subjected to GSEA.

### Gene set enrichment analysis

We utilized the fgsea package (v.1.30.0) in R in combination with the C5 Gene Ontology gene set to determine the pathways that were enriched or downregulated in accordance to m.1227G>A heteroplasmy. Specifically, we calculated the gene-level Spearman correlations between gene expression and m.1227G>A. We then sorted the resulting coefficients in decreasing order to produce a ranked gene list to be used in the fgsea.

### Single-cell m.1227G>A heteroplasmy threshold analysis

To determine the heteroplasmic threshold at which the m.1227G>A variant elicits a phenotype, we calculated the thresholds at which the correlation with gene expression is the most pronounced. To do so, we binned cells in 10% heteroplasmy increments. Then, for each gene, we calculated the Spearman correlation between gene expression and heteroplasmy for every cell greater than the threshold and lower than threshold. As before, we filter out cells that have dropout and only compute the correlation coefficient if at least 100 cells have nonzero gene expression. After Fisher *Z*-transforming the correlation coefficients, we take the absolute delta between the ‘upper’ and ‘lower’ coefficients within each bin.

### Statistics and reproducibility

No statistical method was used to predetermine sample size and no data were excluded from this analysis. The experiments were not randomized and the investigators were not blinded to allocation and outcome assessment. All statistical analyses were performed using R (v.4.1.1). Tests comparing distributions were performed using wilcox.test or aov. Tests comparing proportions were conducted with fisher.test. All statistical analyses were two-sided, unless otherwise specified. When multiple comparisons were performed, *P* values were Benjamini–Hochberg corrected. Figures were produced using ggplot2 and pixel pushed in Adobe Illustrator.

### Reporting summary

Further information on research design is available in the Nature Portfolio Reporting Summary linked to this article.

## Online content

Any methods, additional references, Nature Portfolio reporting summaries, source data, extended data, supplementary information, acknowledgements, peer review information; details of author contributions and competing interests; and statements of data and code availability are available at 10.1038/s41588-025-02374-0.

## Supplementary information

Reporting Summary

Supplementary Tables 1–5 Supplementary Tables 1–5, combined into a single workbook.

## Source data

Source Data Fig. 3 Unprocessed western blots for Fig. 3.

Source Data Fig. 3 Statistical source data for Fig. 3.

Source Data Extended Data Fig. 7 Statistical source data for Extended Data Fig. 7.

## Data Availability

The datasets supporting the conclusions of this article are included within the article and in the Source data. Access to the 100,000 Genomes Project database is available through Genomics England: https://www.genomicsengland.co.uk/. Access to the Helix Mitochondrial Database is publicly available: https://www.helix.com/pages/mitochondrial-variant-database. All reagents used in this study are either commercially available or can be made available from the corresponding authors upon request. Raw metabolomic data were uploaded to MassIVE (MSV000096292) and proteomic data were uploaded to PRIDE (PXD057390). Raw single-cell data are available via Zenodo at 10.5281/zenodo.15367352 (ref. ^47^). Source data are provided with this paper.

## Code availability

Scripts used to regenerate Figs. 1–4 and Extended Data Figs. 1–4 and 8 are available via GitHub at https://github.com/reznik-lab/rrna-hotspots with the relevant data and instructions to execute the code.
