# Supplementary material for: Functionally dominant hotspot mutations of mitochondrial ribosomal RNA genes in cancer
Source: Nat Genet. 2025 Nov 3;57(11):2705–14. doi: 10.1038/s41588-025-02374-0 (PMC12597822; doi:10.1038/s41588-025-02374-0)
Supplement: Supplementary file 1 — Reporting Summary [file 41588_2025_2374_MOESM1_ESM.pdf]

Corresponding author(s): Ed Reznik
Payam Gammage

Last updated by author(s): Sep 15, 2025

Reporting Summary

Nature Portfolio wishes to improve the reproducibility of the work that we publish. This form provides structure for consistency and transparency in reporting. For further information on Nature Portfolio policies, see our Editorial Policies and the Editorial Policy Checklist.

Statistics

For all statistical analyses, confirm that the following items are present in the figure legend, table legend, main text, or Methods section.

n/a	Confirmed	
☐	☒	The exact sample size (*n*) for each experimental group/condition, given as a discrete number and unit of measurement
☐	☒	A statement on whether measurements were taken from distinct samples or whether the same sample was measured repeatedly
☐	☒	The statistical test(s) used AND whether they are one- or two-sided<br>*Only common tests should be described solely by name; describe more complex techniques in the Methods section.*
☐	☒	A description of all covariates tested
☐	☒	A description of any assumptions or corrections, such as tests of normality and adjustment for multiple comparisons
☐	☒	A full description of the statistical parameters including central tendency (e.g. means) or other basic estimates (e.g. regression coefficient) AND variation (e.g. standard deviation) or associated estimates of uncertainty (e.g. confidence intervals)
☐	☒	For null hypothesis testing, the test statistic (e.g. *F*, *t*, *r*) with confidence intervals, effect sizes, degrees of freedom and *P* value noted<br>*Give P values as exact values whenever suitable.*
☒	☐	For Bayesian analysis, information on the choice of priors and Markov chain Monte Carlo settings
☒	☐	For hierarchical and complex designs, identification of the appropriate level for tests and full reporting of outcomes
☒	☐	Estimates of effect sizes (e.g. Cohen's *d*, Pearson's *r*), indicating how they were calculated

Our web collection on statistics for biologists contains articles on many of the points above.

Software and code

Policy information about availability of computer code

Data collection	Western blots were imaged using the Li-Cor Odyssey CLx with ImageStudio (v.5.2).<br>ddPCR data was collected using the BioRad digital droplet PCR system with QX Manager Software Standard Edition (v.2.1).<br>Metabolite abundances were measured using a Q Exactive Orbitrap mass spectrometer coupled to an Ultimate 3000 HPLC (ThermoFisher).<br>Proteomics was performed using the EASY-nLC II 1200 (Thermofisher) coupled to an Orbitrap Fusion Lumos mass spectrometer (ThermoFisher) as part of nanoscale C18 reverse-phase liquid chromatography.<br>single-cell RNA sequencing was performed using a Chromium Next GEM system (10X) coupled to Illumina (Novaseq X+) NGS workflow.
Data analysis	All patient and single cell data was analysed using R (v.4.4.1). All code is publically available at https://github.com/reznik-lab/rrna-hotspots.git.<br>Metabolomic data was analysed using Skyline (MacCoss) and processed using Prism (GraphPad, v9).<br>Proteomic data was analysed using MaxQuant software (v.1.6.1.4) and Perseus software (v.1.6.13.0).<br>Figures were constructed in Adobe Illustrator (Adobe, v.2024).

For manuscripts utilizing custom algorithms or software that are central to the research but not yet described in published literature, software must be made available to editors and reviewers. We strongly encourage code deposition in a community repository (e.g. GitHub). See the Nature Portfolio guidelines for submitting code & software for further information.

Data

Policy information about availability of data

All manuscripts must include a data availability statement. This statement should provide the following information, where applicable:

- Accession codes, unique identifiers, or web links for publicly available datasets
- A description of any restrictions on data availability
- For clinical datasets or third party data, please ensure that the statement adheres to our policy

Genomics data are available within the Genomics England Research Environment, a secure cloud workspace.
Details on how to access data for this publication can be found at https://re-docs.genomicsengland.co.uk/pan_cancer_pub/.
Raw metabolomic data were uploaded to MassIVE (MSV000096292), proteomic data were uploaded to PRIDE (PXD057390) and single cell data were uploaded to Zenodo (15367353).

Research involving human participants, their data, or biological material

Policy information about studies with human participants or human data. See also policy information about sex, gender (identity/presentation), and sexual orientation and race, ethnicity and racism.

Reporting on sex and gender	No human participant data was collected as part of this study.
Reporting on race, ethnicity, or other socially relevant groupings	NA
Population characteristics	Our dataset is comprised 57% female and 43% male patients. The median age is 72 years.
Recruitment	N/A
Ethics oversight	This study uses publicly accessible, anonymized patient sequencing data from several consortia (GEL, TCGA, PCAWG, Helix). This study did not directly recruit or collect data from human participants and therefore was not subject to specific ethical approvals.

Note that full information on the approval of the study protocol must also be provided in the manuscript.

Field-specific reporting

Please select the one below that is the best fit for your research. If you are not sure, read the appropriate sections before making your selection.

☒ Life sciences ☐ Behavioural & social sciences ☐ Ecological, evolutionary & environmental sciences

For a reference copy of the document with all sections, see nature.com/documents/nr-reporting-summary-flat.pdf

Life sciences study design

All studies must disclose on these points even when the disclosure is negative.

Sample size	16,322 total tumor samples were sequenced as part of the 100,000 genomes project. In patients with multiple samples, only the primary tumor sample with the highest tumor purity was retained to yield a total of 14,079 total samples. For wet lab experiments, sample sizes were not predetermined, but followed standard
Data exclusions	Cancer types with less than 3 patients were excluded. No other data were excluded.
Replication	Wet lab experiments were repeated multiple times with similar results, as described in the text.
Randomization	No randomization was employed.
Blinding	Samples for metabolomic, proteomic and scRNAseq analyses were blinded to the operators.

Reporting for specific materials, systems and methods

We require information from authors about some types of materials, experimental systems and methods used in many studies. Here, indicate whether each material, system or method listed is relevant to your study. If you are not sure if a list item applies to your research, read the appropriate section before selecting a response.

Materials & experimental systems

n/a	Involved in the study	
☐	☒	Antibodies
☐	☒	Eukaryotic cell lines
☒	☐	Palaeontology and archaeology
☒	☐	Animals and other organisms
☒	☐	Clinical data
☒	☐	Dual use research of concern
☒	☐	Plants

Methods

n/a	Involved in the study	
☒	☐	ChIP-seq
☒	☐	Flow cytometry
☒	☐	MRI-based neuroimaging

Antibodies

Antibodies used

Total OXPHOS Human WB antibody cocktail (Abcam Cat# ab110411) 1:1000 dilution
MRPS14 (Sigma-Aldrigh Cat# HPA051087) 1:2000 dilution
MRPS27 (Proteintech Cat# 17280-1-AP) 1:2000 dilution
MRPS34 (Proteintech Cat# 15166-1-AP) 1:2000 dilution
MRPL44 (Proteintech Cat# 16394-1-AP) 1:2000 dilution
Goat anti-Rabbit, HRP conjugated (Molecular Probes Cat# G21234) 1:10000 dilution
Goat anti-Mouse, HRP conjugated (Jackson ImmunoResearch Cat# 115-035-146) 1:10000 dilution
Donkey anti-Mouse, IgG Secondary Antibody (Li-Cor, Cat# 926-32212) 1:5000 dilution

Validation

These are all widely used, extensively verified and commercially available antibodies that were purchased directly from the supplier. No antibodies were raised for use in this study.

Eukaryotic cell lines

Policy information about cell lines and Sex and Gender in Research

Cell line source(s)

143B (ATCC), HEK293T (ATCC)

Authentication

All cells were authenticated by in house authentication service through morphology and STR profiling.

Mycoplasma contamination

All cells were tested repeatedly for mycoplasma, and were negative.

Commonly misidentified lines
(See ICLAC register)

Name any commonly misidentified cell lines used in the study and provide a rationale for their use.

Plants

Seed stocks

Report on the source of all seed stocks or other plant material used. If applicable, state the seed stock centre and catalogue number. If plant specimens were collected from the field, describe the collection location, date and sampling procedures.

Novel plant genotypes

Describe the methods by which all novel plant genotypes were produced. This includes those generated by transgenic approaches, gene editing, chemical/radiation-based mutagenesis and hybridization. For transgenic lines, describe the transformation method, the number of independent lines analyzed and the generation upon which experiments were performed. For gene-edited lines, describe the editor used, the endogenous sequence targeted for editing, the targeting guide RNA sequence (if applicable) and how the editor was applied.

Authentication

Describe any authentication procedures for each seed stock used or novel genotype generated. Describe any experiments used to assess the effect of a mutation and, where applicable, how potential secondary effects (e.g. second site T-DNA insertions, mosiacism, off-target gene editing) were examined.
