# Supplementary material for: Functionally dominant hotspot mutations of mitochondrial ribosomal RNA genes in cancer
Source: Nat Genet. 2025 Nov 3;57(11):2705–14. doi: 10.1038/s41588-025-02374-0 (PMC12597822; doi:10.1038/s41588-025-02374-0)

Source Data - Fig. 3

Figure 3G

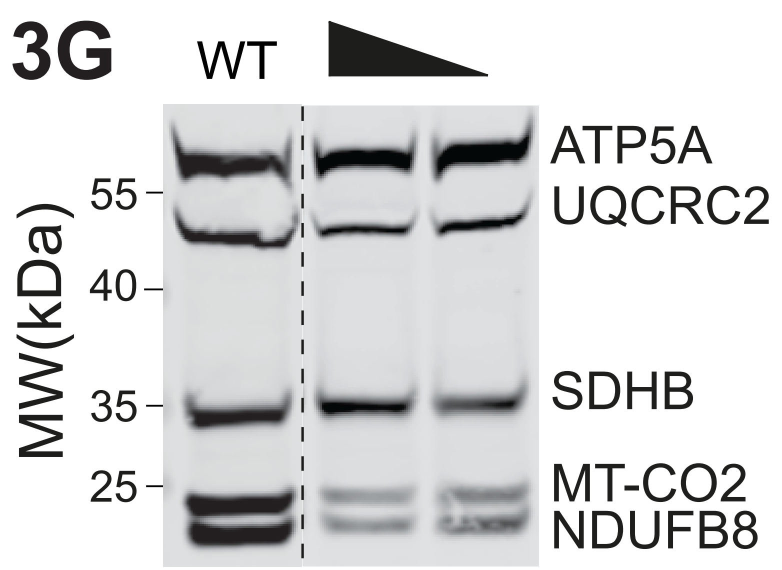

143B WT & 143B m.1227G>A
OXPHOS antibody cocktail

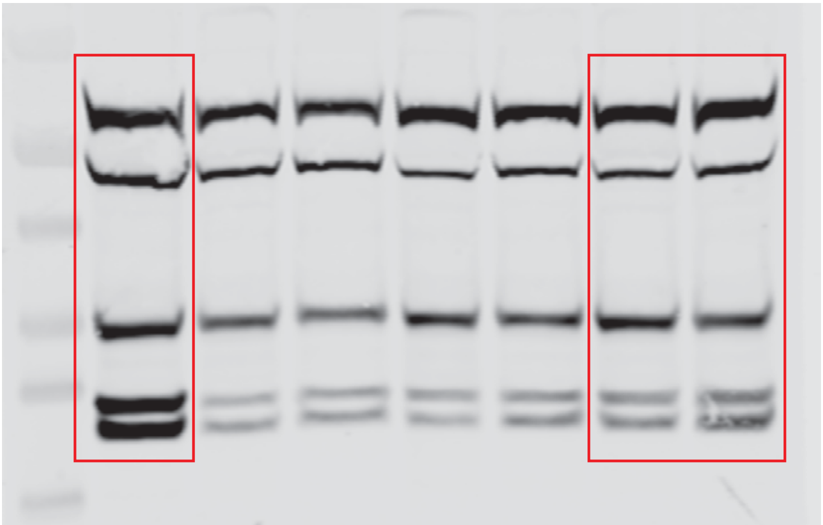

Figure 3H

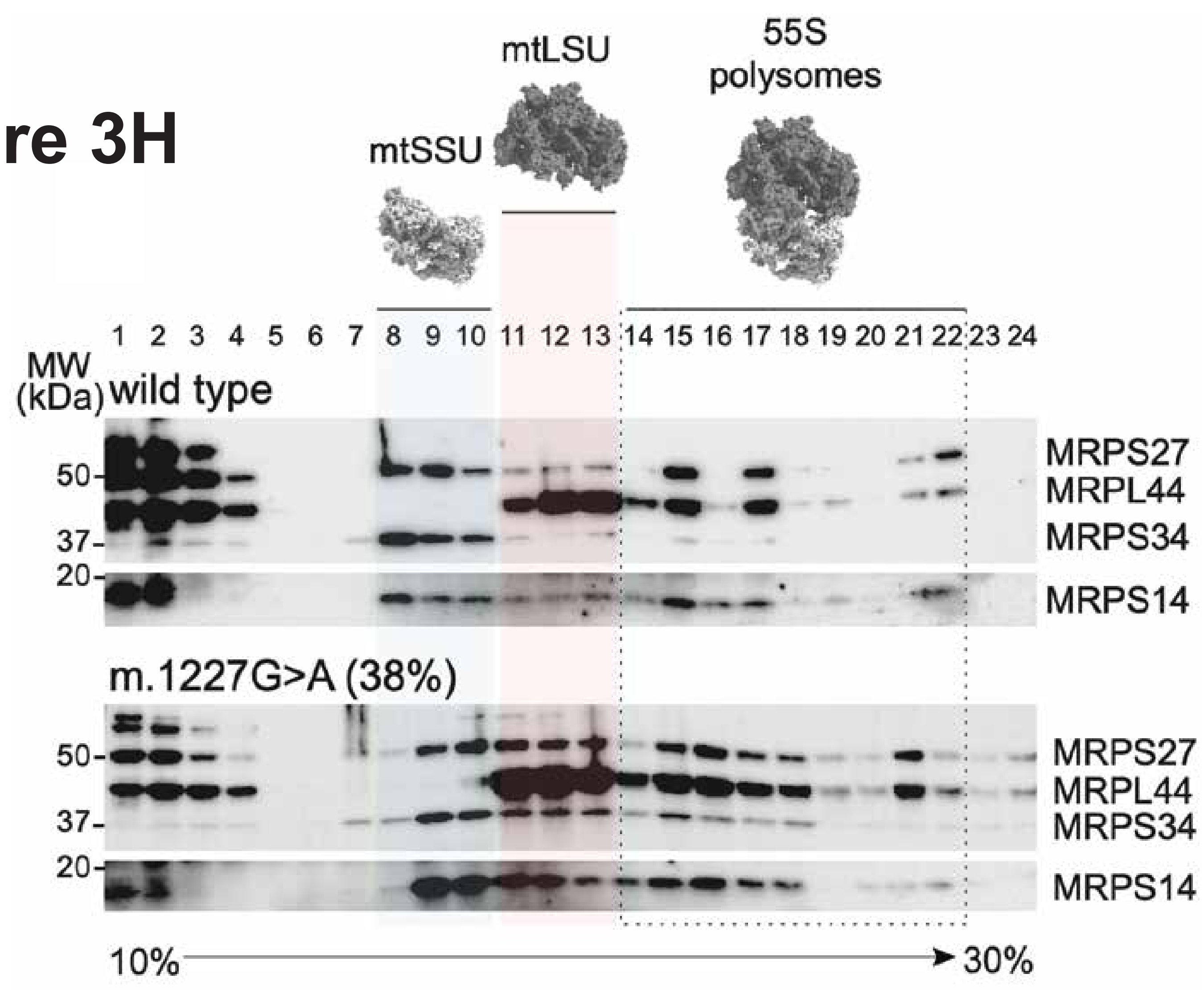

143B WT Sucrose Gradient
MRPS27, MRPL44, MRPS34

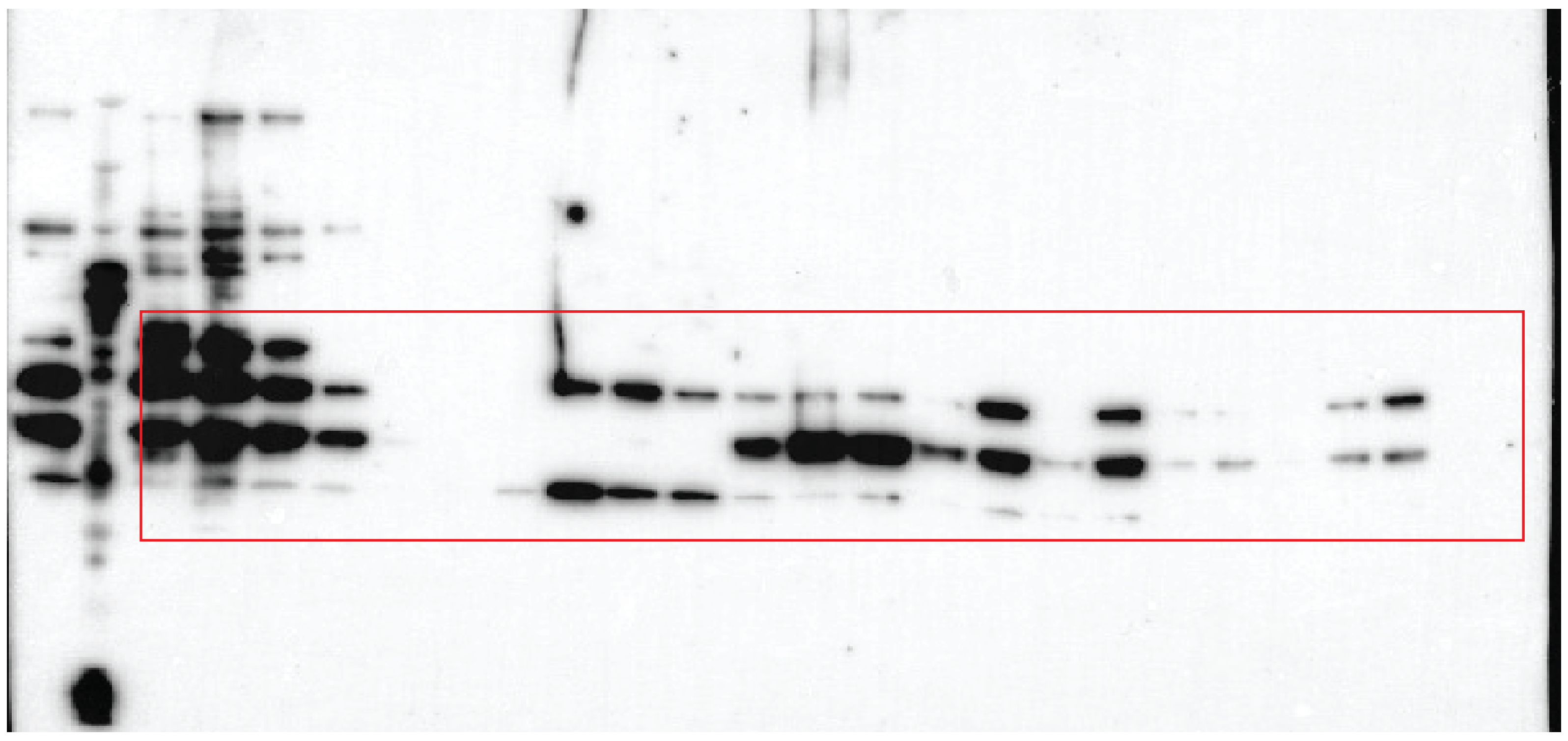

143B m.1227G>A Sucrose Gradient
MRPS27, MRPL44, MRPS34

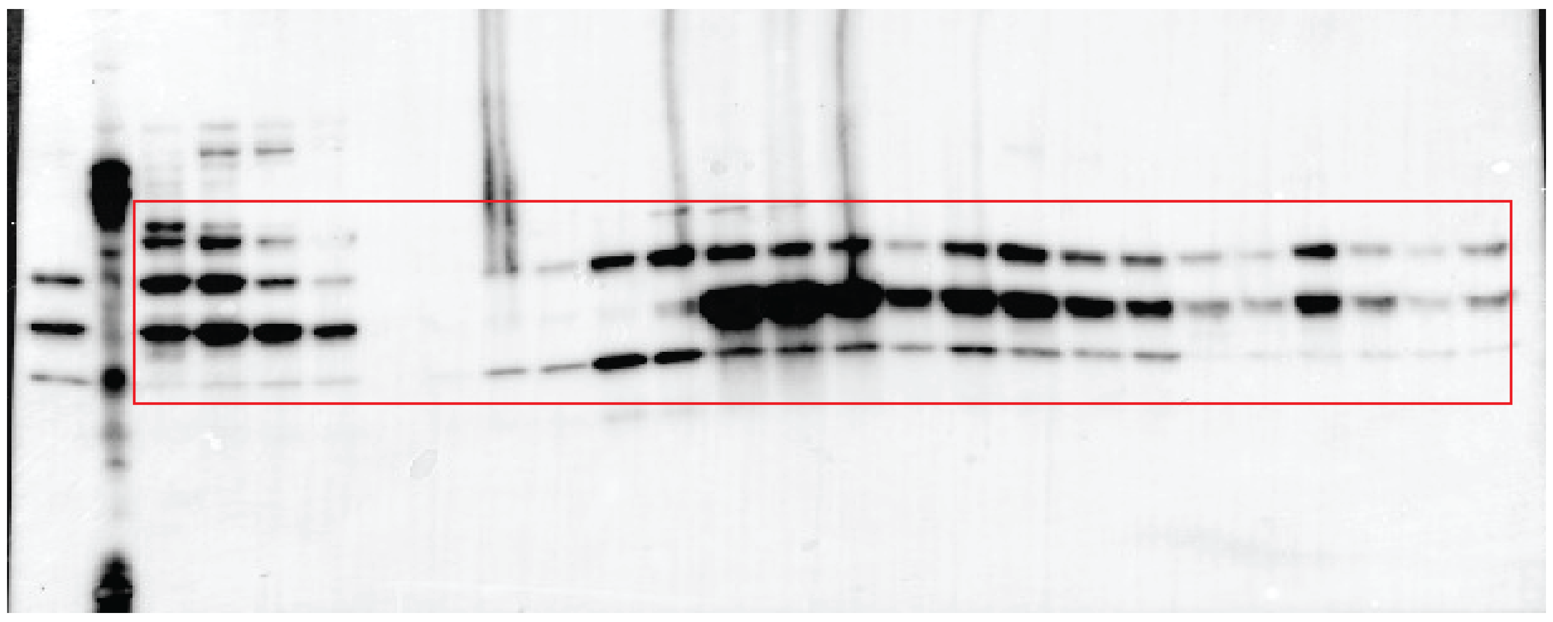

143B WT Sucrose Gradient
MRPS14

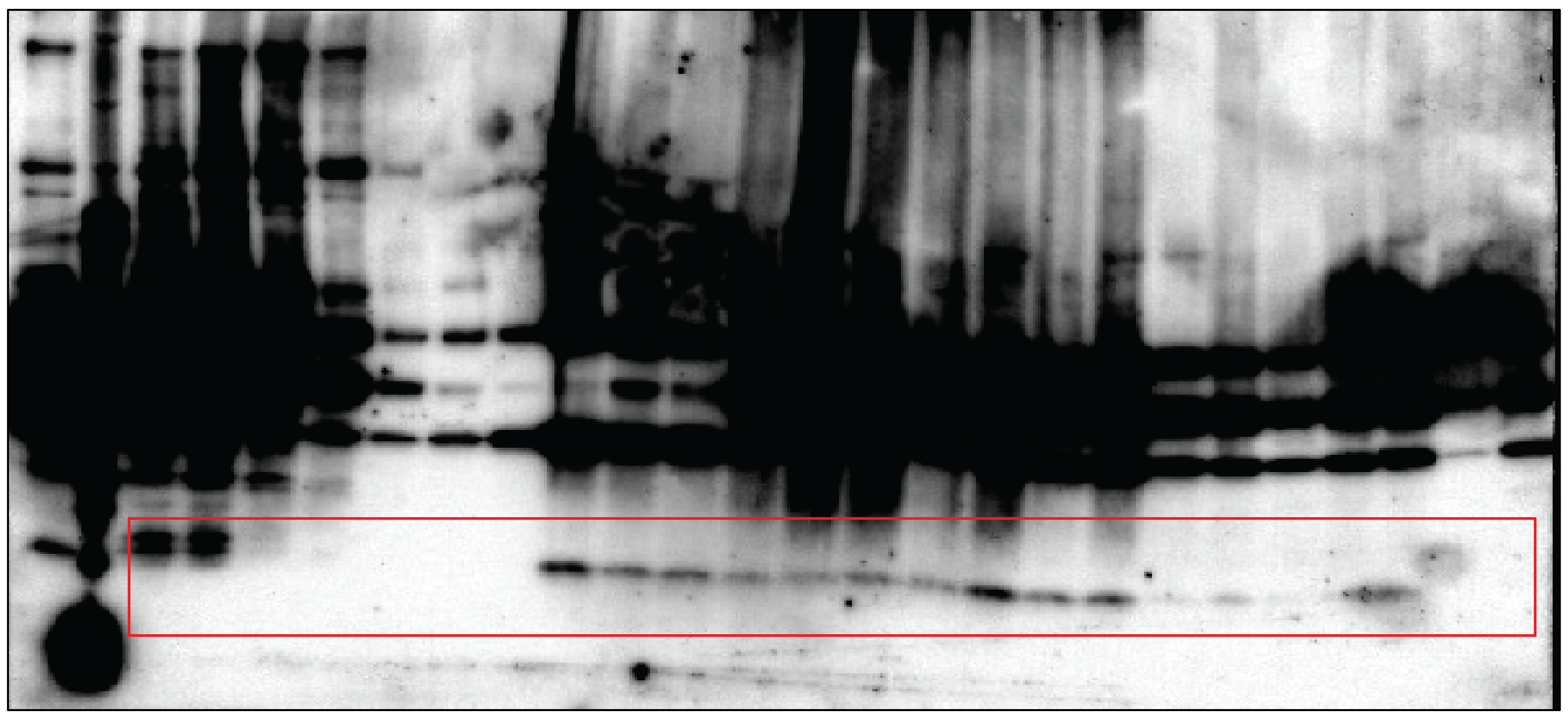

143B m.1227G>A Sucrose Gradient
MRPS14

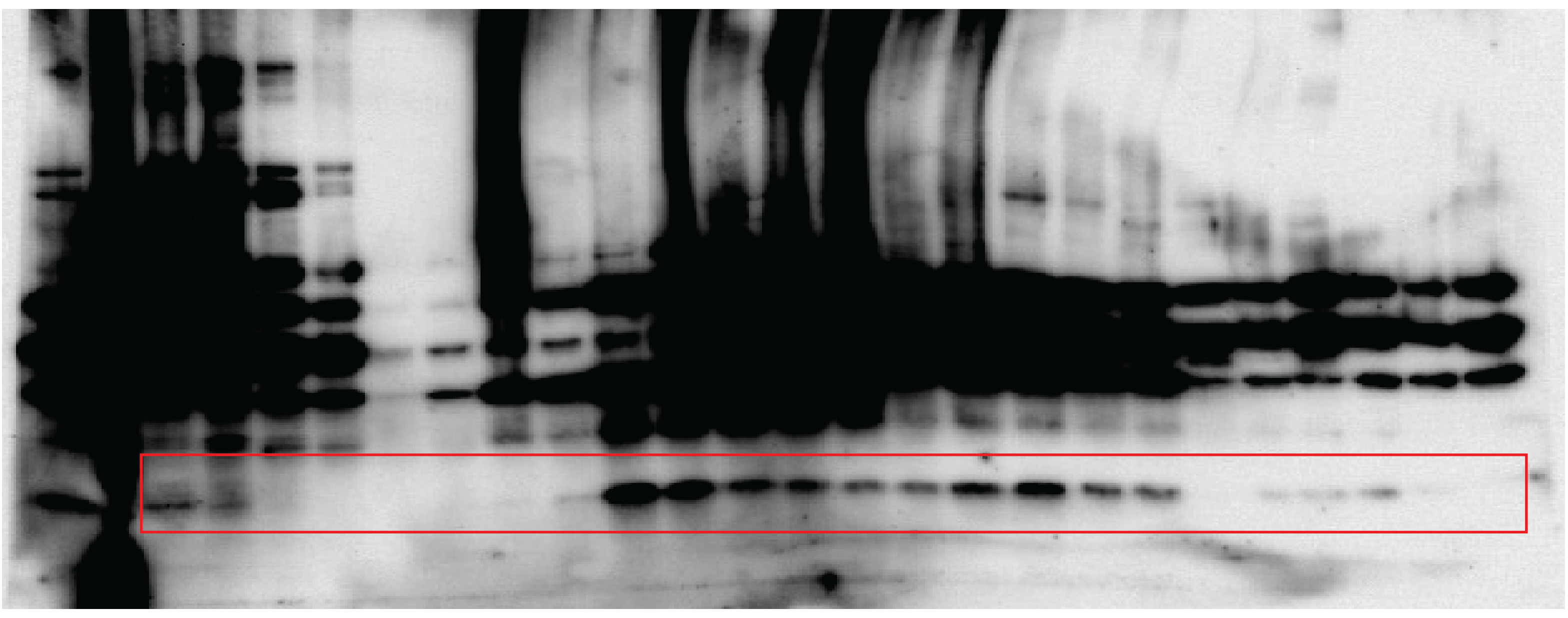

Supplement: Supplementary file 3 — Unprocessed western blots for Fig. 3. [file 41588_2025_2374_MOESM3_ESM.pdf]
